# Multiplexed near infrared fluorescence lifetime imaging in turbid media

**DOI:** 10.1117/1.JBO.29.2.026004

**Published:** 2024-02-29

**Authors:** Meital Harel, Uri Arbiv, Rinat Ankri

**Affiliations:** Ariel University, Department of Physics, Faculty of Natural Science, Ariel, Israel

**Keywords:** fluorescence lifetime imaging, multiplexing, scattering medium, phasor analyses, Monte Carlo simulations, single-photon avalanche diode array, near-infrared imaging

## Abstract

**Significance:**

Fluorescence lifetime imaging (FLI) plays a pivotal role in enhancing our understanding of biological systems, providing a valuable tool for non-invasive exploration of biomolecular and cellular dynamics, both *in vitro* and *in vivo*. Its ability to selectively target and multiplex various entities, alongside heightened sensitivity and specificity, offers rapid and cost-effective insights.

**Aim:**

Our aim is to investigate the multiplexing capabilities of near-infrared (NIR) FLI within a scattering medium that mimics biological tissues. We strive to develop a comprehensive understanding of FLI’s potential for multiplexing diverse targets within a complex, tissue-like environment.

**Approach:**

We introduce an innovative Monte Carlo (MC) simulation approach that accurately describes the scattering behavior of fluorescent photons within turbid media. Applying phasor analyses, we enable the multiplexing of distinct targets within a single FLI image. Leveraging the state-of-the-art single-photon avalanche diode (SPAD) time-gated camera, SPAD512S, we conduct experimental wide-field FLI in the NIR regime.

**Results:**

Our study demonstrates the successful multiplexing of dual targets within a single FLI image, reaching a depth of 1 cm within tissue-like phantoms. Through our novel MC simulation approach and phasor analyses, we showcase the effectiveness of our methodology in overcoming the challenges posed by scattering media.

**Conclusions:**

This research underscores the potential of NIR FLI for multiplexing applications in complex biological environments. By combining advanced simulation techniques with cutting-edge experimental tools, we introduce significant results in the non-invasive exploration of biomolecular dynamics, to advance the field of FLI research.

## 1 Introduction

Fluorescence-based imaging is a powerful tool for *in vitro* and *in vivo* studies. *In vivo* fluorescence imaging (FI) enables to study biomolecules and cell behavior in their native environment. These advantages have been showcased in many biomedical investigations, including probing tissue physiology,^1^ detecting early stages of disease,^2^ sensing molecular concentrations of delivered pharmaceuticals,^3^ observing intracellular fluorescent proteins,^4^^,^^5^ and much more. However, disadvantages of current *in vivo* FI methods include the low penetration of visible light, which is the electromagnetic regime in which most fluorophores emit their fluorescence, through tissue and background autofluorescence. These can be overcome using near-infrared (NIR, or longer) light in biological research or clinical models. Owing to advances in reducing photon scattering, light absorption, and autofluorescence, NIR fluorescence affords higher imaging resolution with increasing tissue penetration depths.

Live animals imaging requires wide-field (parallel) data acquisition, as it minimizes the temporal delay between data recorded in different regions of the field of view. It also has the advantage of dispensing with costly and complex scanning devices, even though it creates other challenges. Single photon avalanche diode (SPAD) camera capabilities for fluorescence lifetime imaging (FLI) microscopy (FLIM) applications in the visible, as well as the NIR, spectrum have been widely described.^6^^–^^8^ SPAD cameras implement a fast time-gating control that are capable of measuring not only the fluctuations of the fluorescence intensity at very high frame rates (50 to 100 kHz)^9^^–^^11^ but also the decay kinetics of fluorophores.^10^^,^^11^ The measurement of fluorescence decay kinetics by each SPAD allows the monitoring of ultra-fast photophysical processes as Förster resonance energy transfer (FRET)^12^ and the identification of multiple fluorescence sources. Although studies demonstrated SPAD cameras’ potential for microscopic biological applications, there are still two main challenges involved with using it for small animal NIR imaging applications: (i) NIR dyes are challenging to detect, due to both the low quantum efficiency of these dyes and the reduced photon detection probability of silicon SPADs in the NIR,^6^ and (ii) most NIR dyes exhibit similar, very short lifetimes, of few hundreds of picoseconds (ps),^13^^,^^14^ which make their detection challenging.

In this work, we investigate the performance of time-gated SPAD arrays in multiplexed wide-field NIR FLI, for further *in vivo* imaging. We present a comprehensive study of the multiplexing capabilities of NIR FLI, using both, simulations and experiments, with a single fluorophore as well as with two adjacent fluorophores behind scattering media that mimic skin tissue with increasing thickness. We first show a novel Monte Carlo (MC) simulation of the path of fluorescent photons in the scattering medium. The MC simulations are a well-known method that allows an in-depth study of the photon path in a turbid medium, such as tissues,^15^^–^^17^ while recent works have extended the MC model to simulate the path of fluorescent photons in biological tissue, reflecting the growing interest in fluorescence-based imaging for *in vivo* biomedical applications.^18^^,^^19^ For example, Welch et al. proposed the most accurate fluorescence MC method,^20^ in which the probabilities of emission and absorption of a photon from a fluorophore are calculated in separate MC codes and later combined by convolution. This method is very time-consuming, although shorter than the direct approach. In this study, we model the photons emitted by a fluorophore within a tissue.^21^ Furthermore, we employ our recently introduced simplified MC model for the propagation of fluorescence photons in biological tissues.^22^ In this model, we disregard excitation photons and treat the fluorophore as an isotropic light source within the tissue. We modeled the time gated camera by a unique, simple process, in which the acquiring gates are presented as time points on the decay curve of the fluorophore. Despite these neglections, which reduce the simulation time by more than half, we show that the general behavior of simulation and experiments correlates well. We then use the results of our simulation method in experimental fluorescence lifetime (FLT) analyses to provide a fast and accurate method for FLI in tissue-like phantoms.

FLI experiments were performed using SPAD512S,^23^ the most advanced large-array SPAD camera, which features a low dark count rate and fully configurable, high-resolution time-gating capabilities that enable FLI with picosecond time resolution and an acquisition time of only 2.6 ms per gate. The resulting data were analyzed using the phasor approach^24^^,^^25^ that provides a user-friendly graphical representation of fluorescence decays while allowing quantitative analysis.

Using our MC simulations, we show the correlation between the quantum efficiency of the fluorophore, its depth in the tissue, and our phasor-based analysis accuracy for extracting its FLT. We also investigate the multiplexing performance of our model as a function of depth in the tissue. We show that the accuracy of FLT calculations for the two fluorophores is significantly improved by applying a simple “cutoff” method to the resulting phasor analyses. These new approaches were applied in our experiments and allowed discrimination between the two NIR dyes in an FLT image behind tissue-like phantoms of 1 cm in thickness.

## 2 Methods

### 2.1 Monte Carlo Simulations

We introduce an MC approach for investigating multiplexed NIR FLI through tissues. Our MC model is rooted in a well-recognized procedure^21^ (see Sec. 3) that pertains to the propagation of photons within biological tissue. We established three core assumptions, strategically designed to streamline and expedite computation time. Selective photon consideration: the model accounts for emitted fluorescent photons only and disregards excitation photons, based on the results from our recent publication.^22^ Temporal emission decay: The number of emitted photons into the tissue diminishes progressively based on a decay constant, intricately linked with the fluorophore’s lifetime. Time-gated acquisition simulation: Photons are assumed to be gathered by the detector at fixed intervals, emulating the gating mechanism characteristic of time-gated acquisition. For simplicity, our approach assigns a gate width (g_w) of zero and a solitary decay encompassing the entire emission process, thus, the accumulation of photons from overlapping gates is not factored in. These assumptions collectively contribute to expediting computation time, while preserving the multiplexed FLI process through tissue within the NIR domain.

**Fig. 1:**
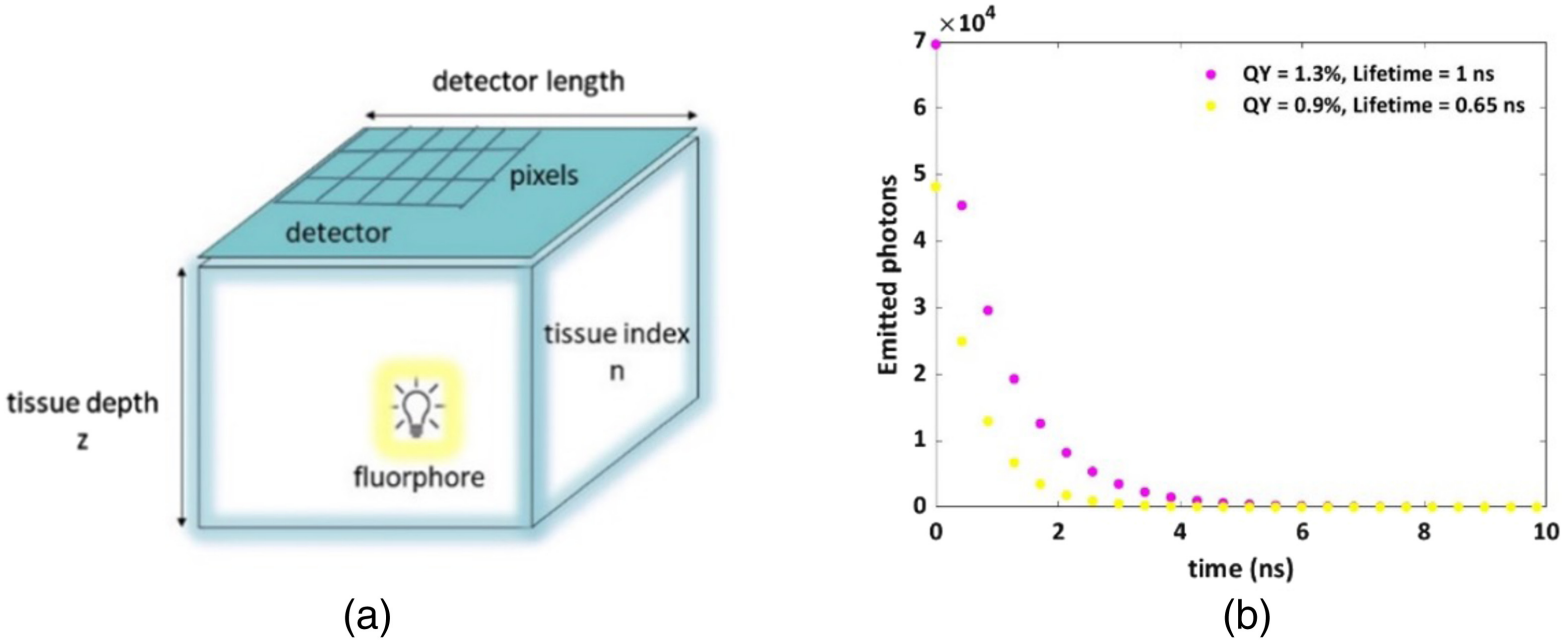
Simulation parameters. (a) A schematic description of the MC model simulating the propagation of fluorescent photons through a tissue of a given thickness z. Parameters used: detector dimensions = 1 × 1 cm, number of pixels = 512*512, PE of the detector = 13%, refractive index n=1.4,^26^^,^^27^ scattering coefficient $\mu s=300\;{\mathrm{cm}}^{-1}$, absorption coefficient $\mu a=0.4\;{\mathrm{cm}}^{-1}$, anisotropy coefficient g=0.96 (for high skin pigmentation,^27^ as well as for piglet,^26^ both in 800 nm in the NIR regime). (b) Emitted photons at specific time intervals, for two fluorophores with the following photophysical characterization: (i) QY = 1.3%; lifetime = 1 ns (mimicking the IRDye800, magenta dots). (ii) QY = 0.9%; lifetime = 0.65 ns (mimicking ICG dye, yellow dots). The time step between each two sample points is g_s = 428 ps, for 117 time samples [only the first 24 time points are shown in Fig. 1(b)]. The initial number of photons, for both fluorophores, was $a=5\cdot 10^{6}$. Emitted photons at specific time intervals, for two other fluorophores used in this paper, are presented in Fig. S1 in the Supplemental Material.

The fluorophore is located at a specific depth, z, in a semi-infinite tissue (infinite in dimensions x and y), with a refractive index n, an absorption coefficient μ_a, a scattering coefficient μ_s, and an anisotropic factor g [see in Fig. 1(a)]. We simulate the photons’ emission as an exponential decay, which correlates to a specific lifetime, under the assumption of an ideal case with no background. Each point in Fig. 1(b) represents a time point at which we sample the emitted photons, with time-intervals of 428 picoseconds (ps, correlated with the experimental time-gating parameters, as will be presented hereinafter) symbolizing a gate step (g_s). The number of emitted photons in each gate step was determined using the fluorophore’s exponential decay:

$$N(t_{\text{emission}})=A\cdot \exp^{-\frac{t_{\text{emission}}}{\mathrm{LT}}},$$ (1)

where A is the pre-exponential factor that consists of the initial number of photons, a, multiplied by the quantum yield (QY) of the fluorophore. Each emitted photon propagated in the tissue [Fig. 1(a)], according to its scattering and absorption properties, for a nonspecific period, then was either absorbed or exited through the tissue surface into the air (n=1). At a given time point, the count was made for all photons reaching the detector, and this count was then multiplied by the photon efficiency (PE) of the single-photon avalanche diode (SPAD) array, set at 13% to match the experimental conditions detailed in Sec. 2.3. Once all photons at a given time point on the decay curve have completed their propagation, the next emission occurred [described by the following point in Fig. 1(b)]. The selection of simulation parameters adhered to the experimental characterization (outlined in the subsequent paragraph) and is outlined in the caption of Fig. 1. Given our focus on simulating emitted photons, and not excitation pulses, the instrument response function (IRF) stemming from the delay between the laser pulse and trigger signal, was deliberately omitted from this simulation, and reduced complexity.

### 2.2 FLT Phasor-Based Analyses

Phasor analyses of the simulated photons, as well as of the experimental time-gated data, were performed as described in our previous paper.^7^ Briefly, the uncorrected, uncalibrated phasor $\left(g_{i,j},s_{i,j}\right)$ of each pixel with coordinate (i,j) in the region of interest (ROI) in the fluorescence image was calculated as follows:

$$\left\{ \begin{array}{c} g_{i,j}=\frac{\sum_{k=1}^{N}F_{i,j}(t_{k})\cos(2\pi kt_{k})}{\sum_{k=1}^{N}F_{i,j}(t_{k})}\\ s_{i,j}=\frac{\sum_{k=1}^{N}F_{i,j}(t_{k})\sin(2\pi kt_{k})}{\sum_{k=1}^{N}F_{i,j}(t_{k})} \end{array},\right.$$ (2)

where f is the phasor harmonic (equal to the laser repetition rate = 1/T), k=1,…,N is the gate number and $F_{i,j}(t_{k})$ is the k’th gate image intensity value at pixel (i,j). The phasor values were calibrated using a known reference sample [in the simulations, the reference was indocyanine green (ICG) with τ=0.65  ns, see Fig. 3(e), and for the experiments we used the IRF lifetime, τ=0]. The modulation and phase were defined as^28^

$$\left\{ \begin{array}{l} \varphi =\tan^{-1}(s/g)\\ m=\sqrt{s^{2}+g^{2}} \end{array}.\right.$$ (3)

The phase lifetime, $\tau_{\varphi }$, was then computed using the following relation:^28^

$$\tau_{\varphi }=\frac{\tan\;\varphi }{2\pi f}.$$ (4)

For each pixel within the ROI, $\tau_{\varphi }$ was computed, furnishing the dataset pivotal for constructing a histogram that displays the frequency of counts corresponding to each $\tau_{\varphi }$ value. In the context of individual dye measurements, the phase lifetimes were calculated by taking the mean value from the histogram. In situations involving multiple dyes, when two adjacent fluorophores occupy the same ROI, the resulting image encompasses two distinct areas, each emitting photons from one of the fluorophores, as well as a third region characterized by a mixture of photons. To effectively delineate these individual regions and consequently discern the original lifetimes, we adopted an approach rooted in outlier exclusion via percentiles, a recognized technique.^6^^,^^7^^,^^27^ This method, termed the “cutoff” method, involves defining a threshold for phasor values resulting from the lifetime histograms. This strategic intervention entails the removal of the extreme values in the phase lifetime outcomes.

### 2.3 Time-Gated Acquisition

The excitation source was a fiber-coupled pulsed laser with a wavelength of 779 nm, 20 MHz repetition rate, and a pulse width of ∼70  ps (VisIR-780, PicoQuant). Wide-field illumination was achieved with a 10× beam expander (BE) (GBE10-B, Thorlabs). The dye solutions were placed between two glass slides separated by a silicon insulator film (Merck Industries, Israel). The emitted fluorescence was recorded with an NIR objective lens (OL) (5018- SW, Computar, Texas) and imaged with the SPAD512S camera (PiImaging, Switzerland). The time-triggered in-pixel architecture enables time-resolved photon counting at a maximum rate of 97 kfps (1-bit frames). The photon detection efficiency of the detector was ∼13% at 800 nm with a fill factor of 10.5% and a dark count rate with a median value of 7.5 Hz/pixel. Overlapping gate images (number of gates; G=117, gate spacing; g_s = 428 ps, gate width; g_w = 10 ns), were used, with a different gate image acquisition time (20 or 50 ms) in each experiment, to account for the different samples’ brightness.

### 2.4 FLT Dyes Solutions Preparation and Photophysical Characterization

IRDye800 NHS (Li-Cor Biosciences, Nebraska) and ICG (Holland-Moran, Israel) were solubilized in phosphate-buffered saline to achieve a final concentration of 100  μM. Subsequently, these solutions were loaded onto glass slides separated by silicon isolator sheets (Merck Industries, Israel). The three-dimensional representations of the excitation and emission spectra for both dyes were acquired using the Fluorolog-QuantaMaster system (Horiba Scientific, Japan). For the collection of lifetime data, the same system was employed, featuring a DeltaDiode-C1 controller (Horiba Scientific, Japan). The samples were subjected to excitation by DeltaDiodes 730L and 830L pulsed lasers (Horiba Scientific, Japan), with peak wavelengths of 730 and 830±10  nm, respectively. Emission signals were detected across wavelengths up to 900 nm. To analyze fluorescence decay curves, we employed the FelixFL decay analysis software (version 1.0.33.0, Horiba Scientific, Japan), which is rooted in a multiexponential model utilizing an iterative reconvolution technique. The fitting quality was evaluated based on the reduced χ2 value, complemented by a visual assessment of the distribution of weighted residuals and their autocorrelation function.

### 2.5 Tissue-Like Phantoms of Varying Thicknesses

Tissue-like phantoms were prepared using a combination of Intralipid (IL) and India ink, subsequently solidified with agarose. In these phantoms, India ink served as the absorbing component, while IL 20% (Lipofundin MCT/LCT 20%, B. Braun Melsungen AG, Germany) was employed as the scattering component.^17^ To ensure consistency, all phantoms contained uniform ink concentration (0.008%) and IL content (0.16%), resulting in an absorption coefficient of $0.04\;{\mathrm{mm}}^{-1}$ and a reduced scattering coefficient of $1.2\;{\mathrm{mm}}^{-1}$, mimicking the optical properties of skin within the NIR range.^26^

The process involved the addition of 1% agarose powder (SeaKem LE Agarose, Lonza) to the solution, to form a gel phantom. The mixture was heated to ∼90°C, with agarose powder slowly integrated while ensuring continuous stirring for optimal uniformity. The resultant blend was subsequently poured into a spacious plastic plate, where it was immersed in water and stored for later use.

For each experimental setup, a slightly oversized slice of the phantom was meticulously carved out and positioned atop a specially designed phantom holder. This holder comprised a glass coverslip affixed to a 3D-printed spacer of precise thickness (ranging from 0.1 to 1 cm). The slice was then cut to achieve the desired thickness and topped with another coverslip for protection (Fig. S2 in the Supplemental Material). These phantom slices were subsequently delicately placed onto the sample, comprising dyes enclosed within a gasket, within the 3D-printed sample holder assembly.

## 3 Results

### 3.1 Simulations

**Fig. 2:**
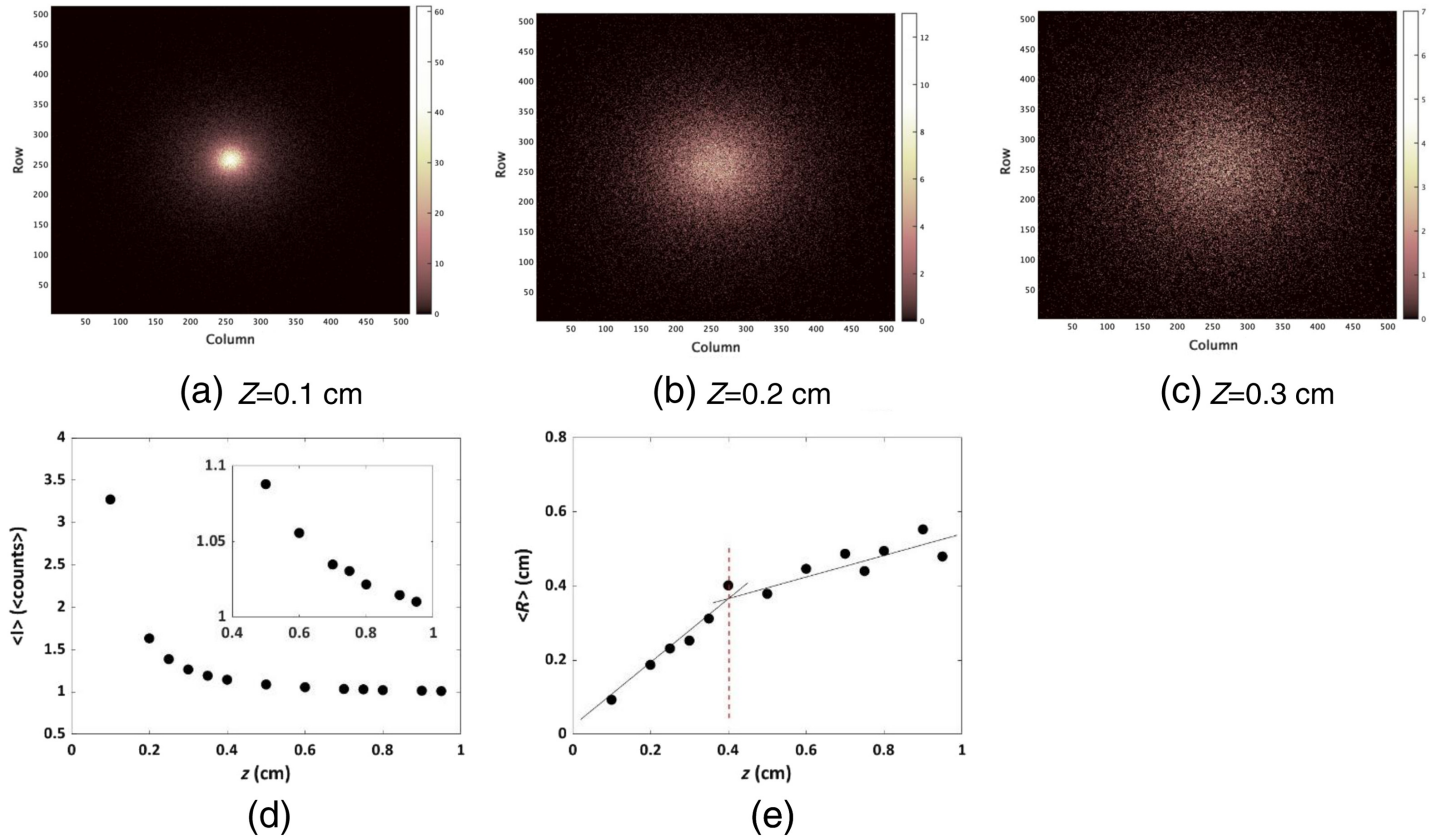
Simulated fluorescence intensity was generated for parameters emulating IRDye800: a lifetime of 1 ns, QY of 1.3%. (a)–(c) These snapshots showcase the distribution of captured photons in a top-down view across different tissue depths z: (a) 0.1 cm, (b) 0.2 cm, and (c) 0.3 cm. The initial number of photons, denoted as $a=7.6\cdot 10^{7}$, was multiplied with fluorophor’s QY (1.3%), resulting with $A=1\cdot 10^{6}$. (d) The average intensity ⟨I⟩ (measured in counts) for different depths z. Inset: An expanded view of ⟨I⟩ plotted versus z, within the depth range of 0.3 to 0.95 cm. (e) The average radius ⟨R⟩ as a function of z. The dashed red line represents the z value in which the dependence of ⟨R⟩ on z values is diminishing.

In Figs. 2(a)–2(c), we show representative results for detector-recorded photons during the simulated recording period, corresponding to various tissue thicknesses z (0.1 to 0.3 cm in thickness). These recordings pertain to the fluorophore emulating IRDye800 with a lifetime of 1 ns. Clearly, the “spots” of intensity exhibit a more pronounced scattering effect as the phantom depth increases. In Fig. 2(d), the average intensity ⟨I⟩ (counts) is plotted against tissue depth z. This average intensity was determined using the following equation:

$$\langle I\rangle =\frac{\sum N_{\text{photons}}}{\sum N_{\text{pixels}}}.$$ (5)

Clearly, there is a discernible decline in intensity with the increasing of z, a consequence attributed to the heightened occurrence of scattering and absorption events. Fig. 2(e) shows the variation in the average radius ⟨R⟩ of these intensity “spots,” for different depths of the tissue z. To calculate this mean radius, each photon’s position was measured from the center of the spot. The mean radius is defined as the sum over all radii, divided by the total number of them:

$$\langle R\rangle =\frac{1}{N}\overset{N}{\underset{i=1}{\sum }}R_{i}.$$ (6)

**Fig. 3:**
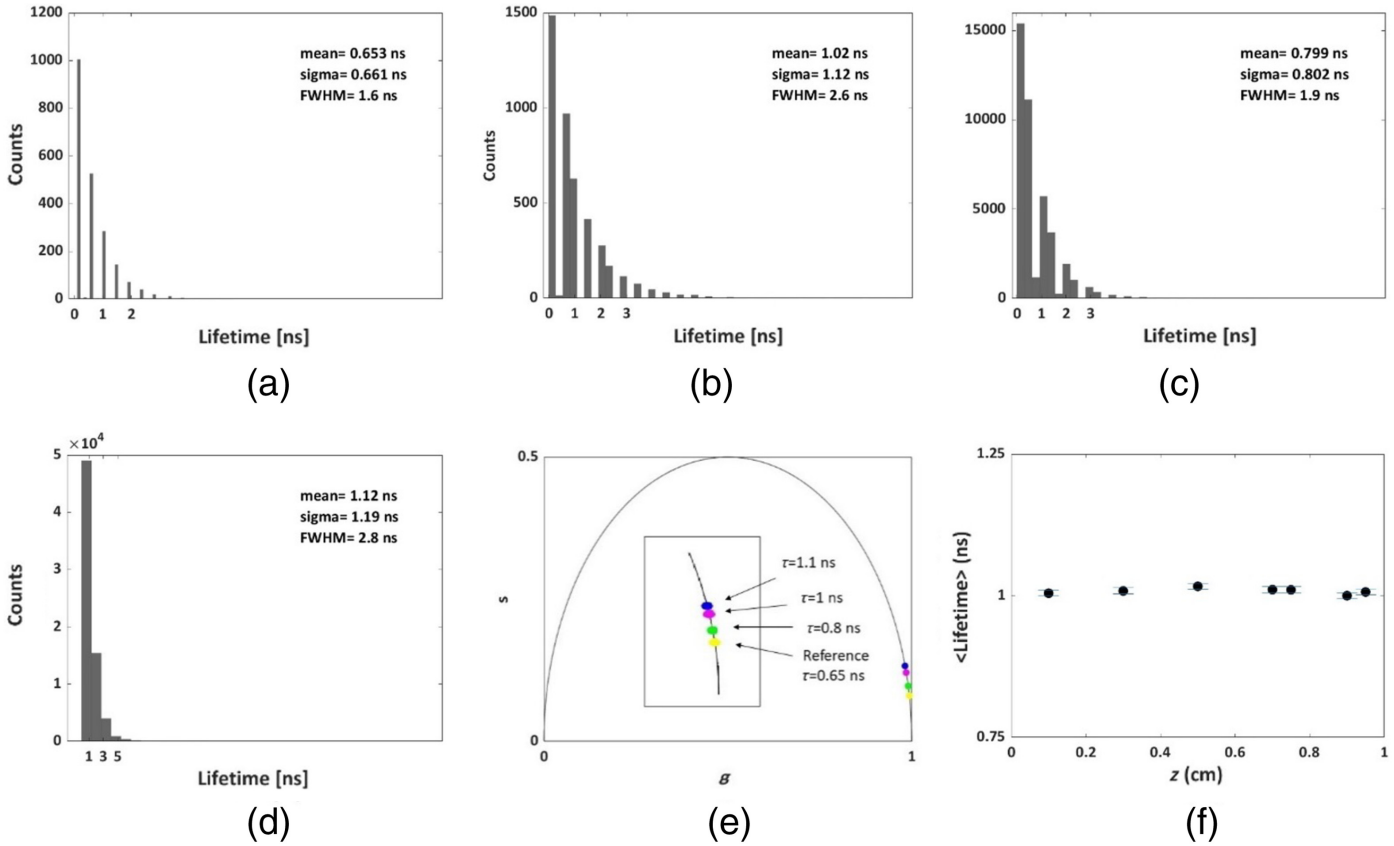
Lifetime analysis of a single fluorophore located at a representative depth of z=0.3  cm within the simulated tissue. (a) QY = 0.9%; lifetime = 0.65 ns. (b) QY = 1.3%; lifetime = 1. (c) QY = 18%; lifetime = 0.8 ns. (d) QY = 28%; lifetime = 1.1 ns. (e) Locating the mean lifetimes extracted from the histograms (a)–(d) on the UC. ICG was set as the reference dye. The initial number of photons for all simulations was $a=5\cdot 10^{6}$. (f) Simulated FLTs extracted for the fluorophore emulating IRDye800 from all tissue depths are shown in Fig. 2. Results show a consistent FLT of 1.00±0.01  ns. The standard deviation of the resulting lifetime for each depth as the deviation from the mean fluorescence lifetime obtained for all depths. Figure S5 in the Supplemental Material displays intensity images corresponding to the data depicted in (a)–(d). These images represent a single fluorophore positioned at a representative depth of z=0.3  cm within the simulated tissue. Figure S6 in the Supplemental Material shows two representative lifetime results which were the foundation for the graph presented in (f).

As anticipated, there is a noticeable enlargement in ⟨R⟩ as the tissue depth increases, attributed to the increase in scattering phenomena. The dependence of ⟨R⟩ on z values is diminishing as depth increases [Fig. 2(e), the dashed red line]. This intriguing trend, displaying a reduced reliance of fluorescence intensity on scattering at greater tissue depths, aligns with recent findings, as was presented by Weber et al.,^22^ which provided a novel diffusion theory for the trajectories of fluorescent photons within turbid media with an adequate explanation for this behavior. Similar outcomes for the average intensity and mean radius for different dyes are accessible in Fig. S3 in the Supplemental Material. These results, showcasing an expected and well-understood pattern, enhance the credibility of our MC model and pave the path for subsequent fluorescence lifetime analysis, elaborated upon below. The behavior of the IRDye800 fluorophore’s FLT at different depths is presented in Fig. 3(f).

Once the precision of our model for wide-field FI through a scattering medium was established, we harnessed the phasor analysis method to deduce a sample’s lifetimes from our simulated data. Figure 3 presents the outcomes; lifetime analyses for four dyes differ by their FLTs (0.65, 1, 0.8, and 1.1 ns) and QYs [(0.9%, 1.3%, 18%, and 28%, Figs. 3(a)–3(d), respectively]. The resulting phasor plots are shown in Fig. 3(e).

Leveraging the procedures outlined in Sec. 2.2, we computed phasor analyses and histograms of lifetimes for all pixels. This histogram encompasses a range of lifetime values attributed to varying pixels within the ROI. In Figs. 3(a)–3(d), the calculated lifetime values are visually portrayed. As our model presents a single exponential decay, alongside gates of zero width (to reduce calculation time), the number of the detected photons was relatively low, giving the lifetime histogram a discrete behavior. Therefore, Figs. 3(a)–3(d) reveal that an increase in the photon counts (corresponding to the increase in the QY of the fluorophore) gives the histogram a more continuous demeanor. Figure 3(e) shows the phasor plot for the mean lifetime values documented in Figs. 3(a)–3(d), while the simulated dye resembling ICG serves as the reference FLT. The observed values are positioned on the semicircle, which aligns with the expected pattern for single exponential lifetimes;^25^ the shorter lifetime is in proximity to the (1,0) coordinate, while longer lifetimes are approaching the (0,0) coordinate, as anticipated.^25^ Figure 3(f) exhibits mean lifetime values for the fluorophore emulating IRDye800 corresponding to diverse depths. The computed mean lifetime versus depth yields a value of 1.00±0.00  ns, across all tissue depths, indicating that our phasor analysis can unveil the FLT of a fluorophore from deep within the tissue. Comparable findings for additional different dyes are available in Fig. S4 in the Supplemental Material.

For further approval for the model and way of calculation, we run the simulation across different optical properties such as an order of magnitude higher scattering coefficient ($\mu s=503\;{\mathrm{cm}}^{-1}$ instead of $\mu s=403\;{\mathrm{cm}}^{-1}$). The results are available in Fig. S7 in the Supplemental Material. Results show that despite the different optical properties, our method enables us to reveal the ground true values of the fluorophores, and therefore contributes to model approval.

**Fig. 4:**
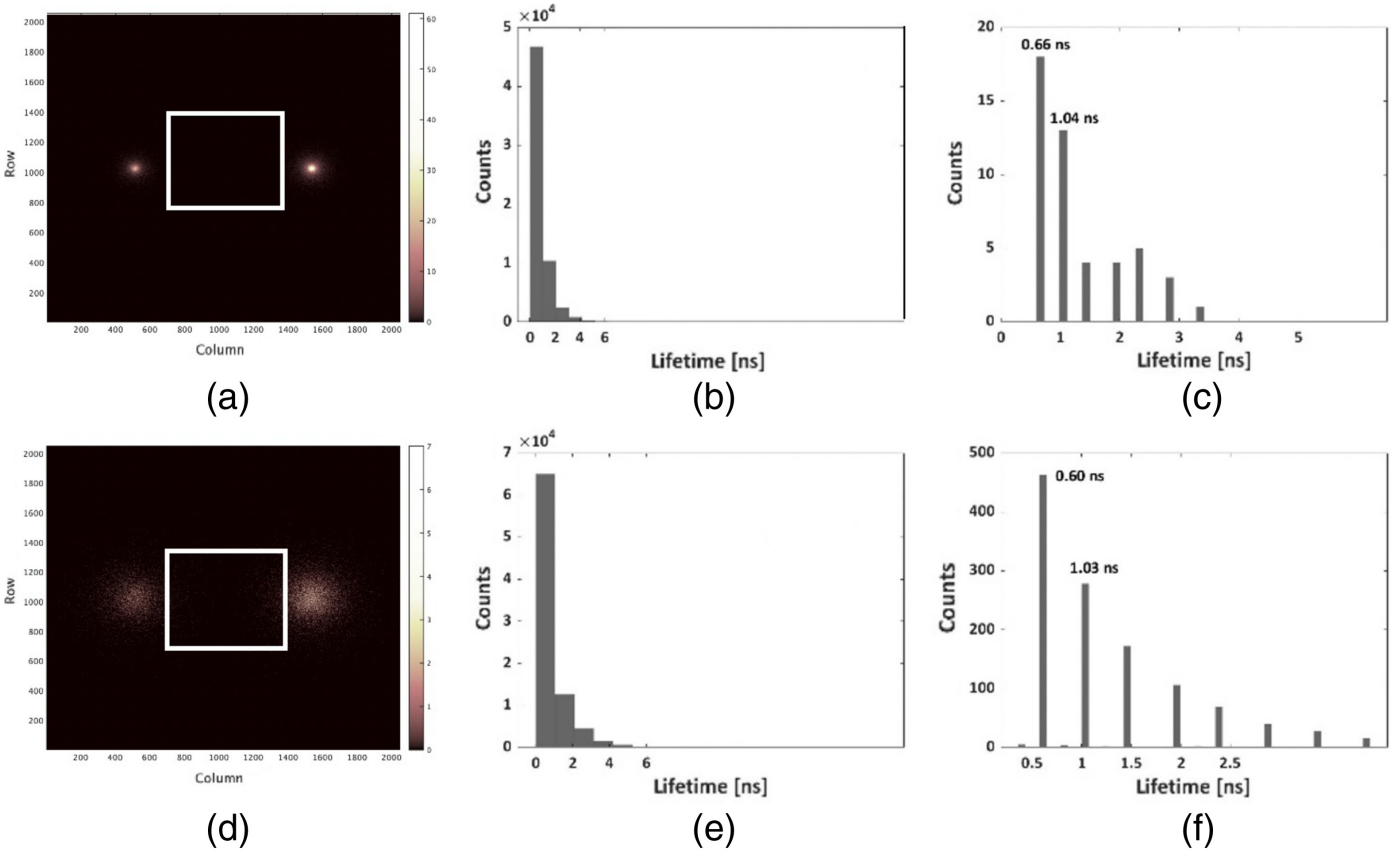
Simulated fluorescence intensity and phasor analysis results pertaining to two adjacent fluorophores: Two adjacent fluorophores (left spot: QY = 0.9%, lifetime = 0.65 ns, right spot: QY = 1.3%, lifetime = 1 ns) from a top-down view. The vertical separation between the centers of these fluorophores was Δx=2  cm, with an initial photons number a of $∼7.6\cdot 10^{7}$. (a) The simulated intensity distribution, for a fluorophore’s depth of 0.1 cm within the tissue. Dimensions are 2048*2048  pixels. The white square designates the ROI where phasor analyses were conducted. (b) The histogram of lifetimes, for the large frame shown in Fig. 1(a). (c) The histogram of lifetimes for the white frame of Fig. 1(a), featuring a cutoff at 1%. The detector’s dimensions are 512*512  pixels. Due to our selection of a minuscule ROI for heightened model rigor, the resulting histogram exhibits a limited number of bars. (d) The simulated intensity distribution for the two fluorophores while they are situated at a tissue depth of z=0.3  cm. Dimensions are 2048*2048  pixels. The white square designates the ROI (512*512  pixels) where phasor analyses were conducted. (e) The histogram of lifetimes for the large frame is illustrated in Fig. 1(d). (f) The histogram of lifetimes for the white frame of Fig. 1(d), featuring a cutoff at 1%. The detector’s dimensions are 512*512  pixels.

After confirming the accuracy of our simulation in extracting FLT for various fluorophores and depths within tissues, we proceeded to a fundamental inquiry in the realm of FLI: its efficacy in discerning between two neighboring fluorophores, based on their distinct lifetimes $\tau_{1}$ and $\tau_{2}$, and for different spatial distances, within a single image. This section embarks on an exploration of simulated phasor-based analyses, aimed at extracting the lifetimes of two proximate fluorophores while varying the spatial gap (Δx), lifetime separations ($\Delta \tau =\tau_{2}-\tau_{1}$), and different tissue thicknesses (z). Figure 4(a) shows a representative simulated intensity image of two adjacent fluorophores (their FLTs and QYs as shown in the Fig. 4 caption), as observed from a top-down view, positioned at a depth of 0.1 cm within the tissue. The vertical separation between the centers of these fluorophores was Δx=2  cm (which is higher than the detector’s original width, which is 0.84 cm, corresponding with 512 pixels of 16.38  μm each).^6^ The designated ROI is depicted by the white rectangle. Figure 4(b) presents the lifetime histograms and phasor analyses for the entire image frame shown in Fig. 4(a), while Fig. 4(c) presents the lifetime histograms and phasor analyses for the chosen ROI. As described in the Sec. 2, when dealing with two adjacent fluorophores within the same ROI, an outlier exclusion method should be employed. This involved setting a threshold for the resultant phasor values within the lifetime histograms, aiming to delineate distinct photon regions and subsequently isolate separate lifetimes. The lifetime histogram featuring a 1% cutoff value was calculated for the specific ROI, and the result is depicted in Fig. 4(c). The two prominent lifetime peaks observed at 0.66 and 1.04 ns correspond to the simulated values of 0.65 and 1 ns, respectively. Without this delineate distinct photon regions, lifetime extraction was not possible. With the encouraging results from our FLT analyses, we proceeded to undertake a more rigorous assessment of our novel model’s capabilities. Figures 4(d)–4(f) show FLI results at greater tissue depth: at z=0.3  cm, indicating that lifetime extraction remains unaffected by increasing scattering events at higher tissue depths. This consistency echoes the conclusions drawn in our earlier work by Ankri et al.^7^ It is noteworthy that despite focusing the analysis on pixels at the edges of the intensity spots, the results exhibit a strong correlation with the predefined fluorophore parameters [Figs. 4(c) and 4(f)]. These observations substantiate the efficacy of our novel MC model in the realm of multiplexing diverse FLT measurements. Results for smaller vertical separation are available in Fig. S8 in the Supplemental Material and highlight the outcomes obtained when reducing the vertical separation to Δx=1  cm, quantified by a vertical line linking the intensity spot centers.

**Fig. 5:**
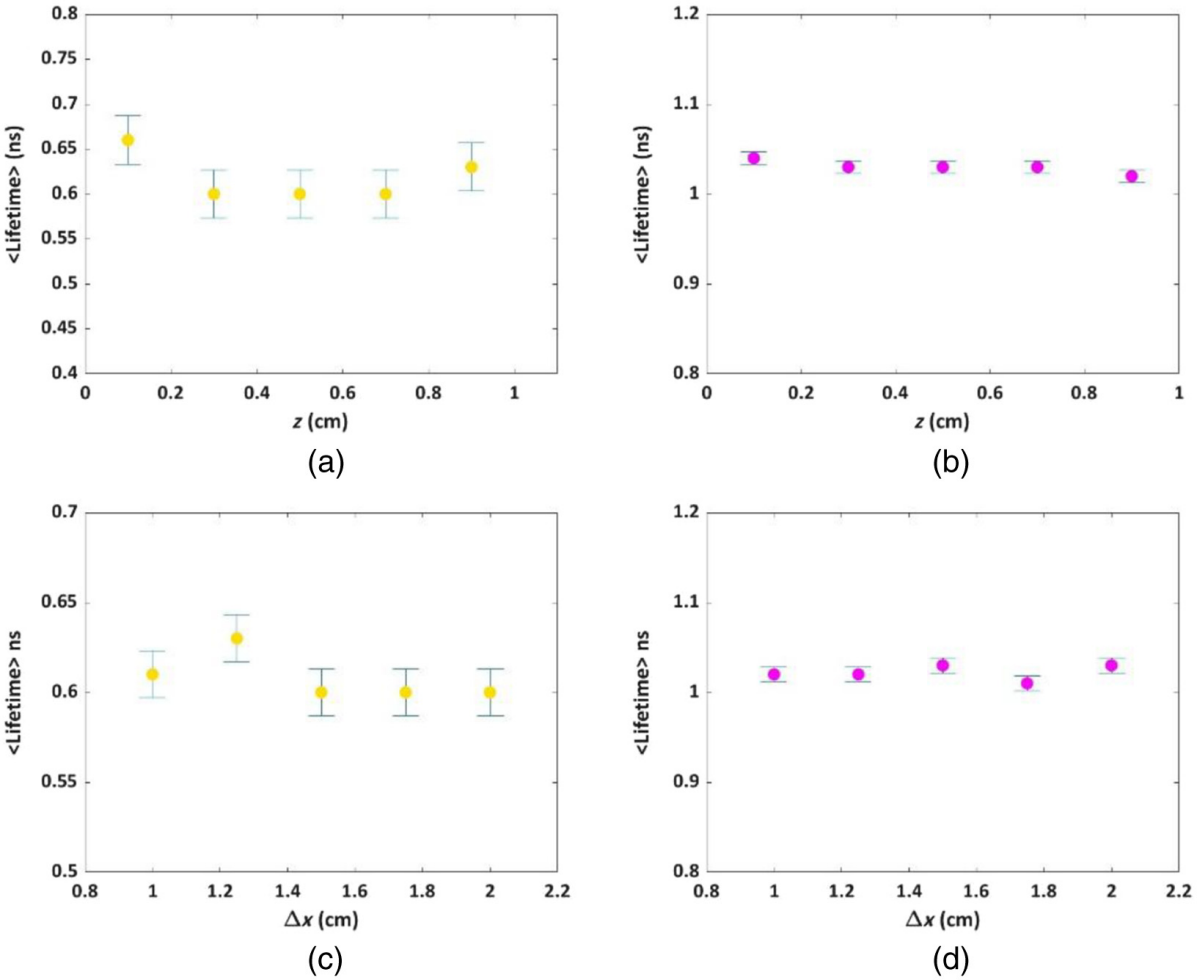
(a), (b) Lifetimes versus depth after 1% cutoff for two adjacent fluorophores at a fixed vertical distance Δx=2  cm. (a) The fluorophore F1 and (b) the fluorophore F2. The mean lifetime value versus depths was 0.61±0.02  ns for F1 – yellow and 1.03±0.00  ns for F2 -magenta. (c), (d) Lifetimes of two adjacent dyes for different vertical distances, Δx. (c) FLTs results for F1. (d) FLTs results for F2. The mean lifetime values versus vertical distance were 0.60±0.04  ns for F1 and 1.04±0.00  ns for F2.

Assessing the limitations inherent in a novel model constitutes a crucial phase in its investigation. Therefore, our subsequent inquiry involved scrutinizing the capacity for multiplexing of our fresh model within increasing depths and shorter distances. Figures 5(a) and 5(b) show a summary of the multiplexing outcomes obtained from the simulated samples’ FLT measurements at various depths (z=0.1, 0.3, 0.5, 0.7, 0.9 cm) following a 1% cutoff. The two distinct fluorophores, namely F1 [yellow, shown in Fig. 5(a)] and F2 [magenta, shown in Fig. 5(b)], were maintained at a constant separation of Δx=2  cm. This spacing data were derived from the information presented in Fig. 4, along with supplementary data found in Fig. S9 in the Supplemental Material. The mean lifetime values with associated uncertainties for F1 and F2 at different depths were 0.61±0.02 and 1.03±0.00  ns, respectively, exhibiting a strong alignment with the FLT values that were originally inserted for all the investigated depths. Figures 5(c) and 5(d) show the determined lifetimes corresponding to vertical separations of the two neighboring fluorophores, F1 and F2: specifically, Δx=1, 1.25, 1.5, 1.75, and 2 cm, while maintaining a consistent depth of z=0.3  cm. This information is compiled from the data showcased in Fig. 4, in conjunction with supplementary details available in Fig. S10 in the Supplemental Material. As for the mean lifetime values pertaining to the vertical distances, they stand at 0.60±0.01  ns for F1 and 1.02±0.00  ns for F2. These findings aptly correspond with the initially incorporated FLT values across all configurations. In the Supplemental Material, we include similar results for additional dyes (Fig. S11 in the Supplemental Material).

### 3.2 Experiments

**Fig. 6:**
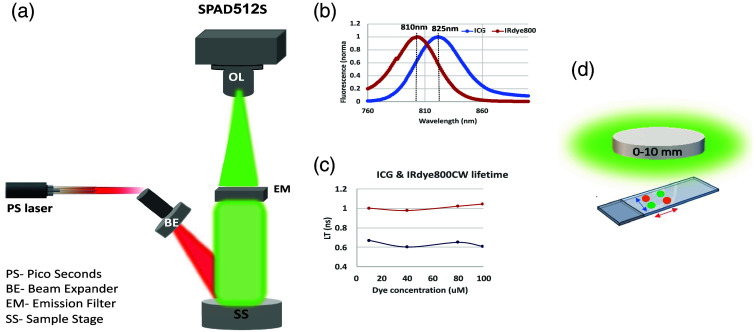
Experimental parameters and setup. (a) Configuration of the experimental arrangement utilized in time-gated FLI: A picoseconds (PS) laser emitting at 779 nm, coupled through a fiber, illuminates the sample employing a wide-field approach with a X10 BE. Fluorescence from the sample stage is collected by an OL and directed toward the SPAD array via an 808 nm long-pass emission filter (IDEX Health & Science, LLC). (b) Emission spectra of the two employed fluorophores in our investigations: IRDye800 (red line) and ICG (blue line). (c) FLT measurements of the two dyes, acquired for varying concentrations of IRDye800 (resulted with an average value of 1 ns, depicted by the red line) and ICG (resulted with an average value of 0.65, illustrated by the blue line). (d) The sample arrangement was assembled on a slide, separated by a silicone gasket. The dyes IRDye800 and ICG (represented in orange and green, respectively) were applied at distances of 0.5 and 1 cm from each other (indicated by blue and red arrows, correspondingly). Multiple phantom layers, differing in thickness (0, 0.3, 0.5, 0.7, and 1 cm), were added onto the slide.

Two commercially available dyes, ICG and IRDye800, each possessing measured lifetimes $\tau_{1}=0.62\pm 0.03$ and $\tau_{2}=1\pm 0.02$, respectively [as shown in Fig. 6(d)], were positioned at two distinct intervals: Δx=0.5 and Δx=1  cm apart from each other. Phasor analyses were performed on each dye separately, as well as for two adjacent dyes, as detailed hereinafter.

**Fig. 7:**
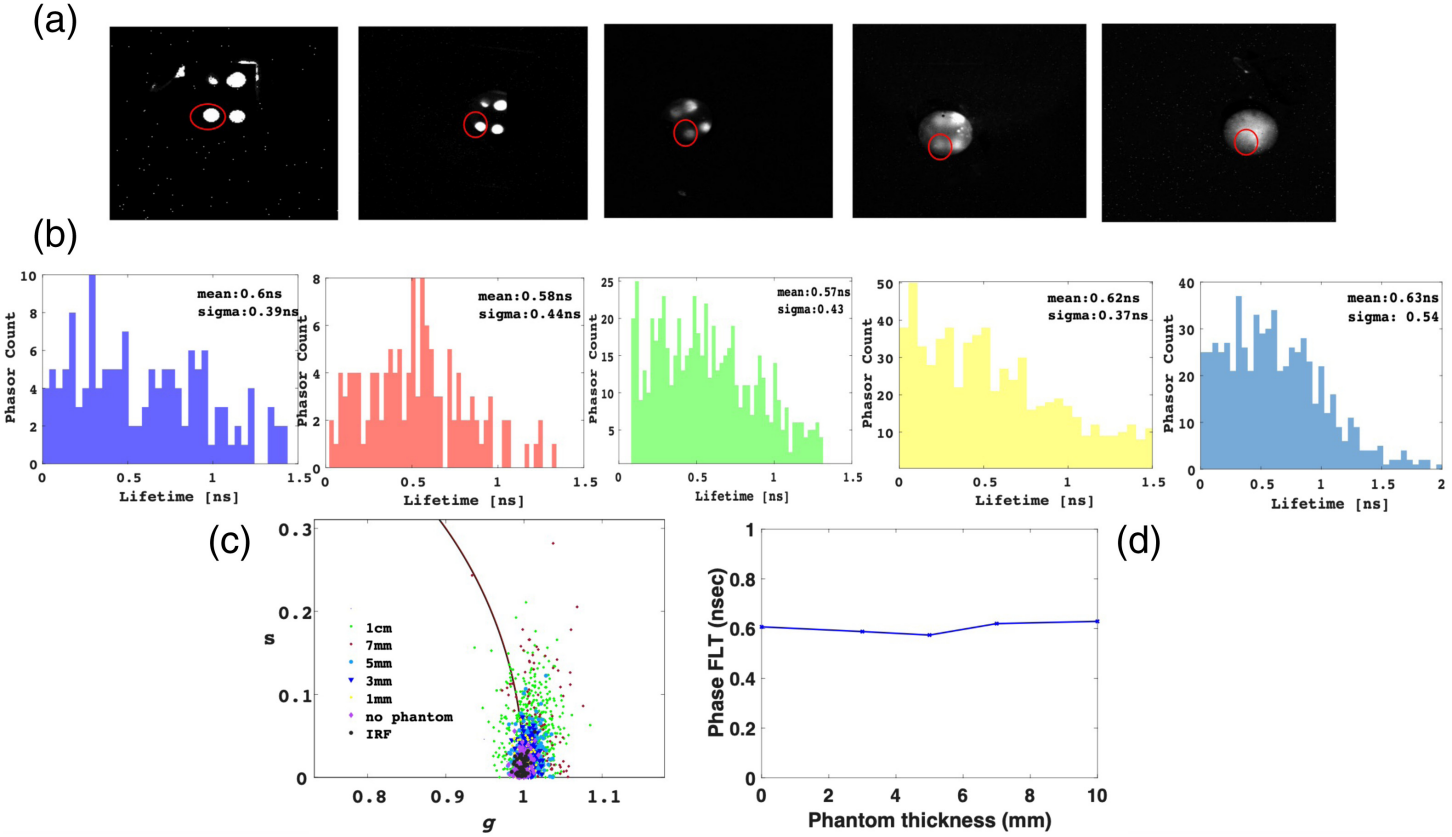
ICG fluorescence lifetime extraction behind IL tissue-like phantoms. (a) Intensity images depicting the dyes within diverse IL phantoms of varying thicknesses, ranging from 0 to 1 cm. The scale bar is 12 mm. (b) Histograms representing the distribution of phasor counts for each calculated lifetime. (c) Phasor “clouds” illustrate the IRF and dyes’ samples. The scattering of the “clouds” increases as the phantom thickness increases. (d) The mean FLT extracted for the ICG dye, as a function of the increasing phantom thickness.

Figure 7(a) shows intensity images of the two fluorophores captured from four distinct locations, with imaging performed through phantoms of varying thicknesses (0, 0.3, 0.5, 0.7, and 1 cm). Notably, as the phantom thickness increases, the images progressively lose clarity, leading to an eventual inability to differentiate between the four distinct dyes when imaged through a phantom that is 1 cm thick. In Figs. 7(b)–7(d), an in-depth analysis is demonstrated for ICG, specifically focusing on the dye enclosed by the red circle in Fig. 7(a). Figure 7(b) portrays phasor “clouds” that have been computed for each pixel within the ROI. Evidently, the scattering of the phasor clouds amplifies as the phantom’s thickness increases, aligning with observations previously documented by our research.^7^ Utilizing these phasor results, we derived histograms detailing the frequency of phasor counts for each FLTs, as shown in Fig. 7(c). Despite the growing dispersion in FLT values, all samples exhibited remarkably similar mean FLT values. This culminated in an average outcome of 0.60±0.02  ns, as shown in Fig. 7(d). The integration of the advanced SPAD512S microlensed detector, combined with pixel-level background correction and phasor-driven analyses, empowered the extraction of the dye’s FLT even when obscured behind a phantom that is 1 cm thick.

Analogous phasor-based analyses were conducted for IRDye800, yielding an average FLT of 1.10±0.06  ns (as depicted in Fig. S13 of the Supplemental Material). This measurement well correlates with the FLTs recorded by our time-correlated single photon counting setup [refer to Fig. 6(c)]. These findings underscore the capability of phasor-based analyses to perform FLI even for tissue-like phantoms as thick as 1 cm, primarily through the utilization of mean average calculations applied to the phasor cloud. For both dyes, there was a slight increase in FLT as the phantom thickness grew, albeit the mean “phase FLTs” remained akin to the established FLT values of 0.65 and 1 ns for ICG and IRDye800, respectively. This observation suggests that despite the relatively low Δτ between the two dyes, their multiplexing can be accomplished through the employment of the phasor-based analyses.

**Fig. 8:**
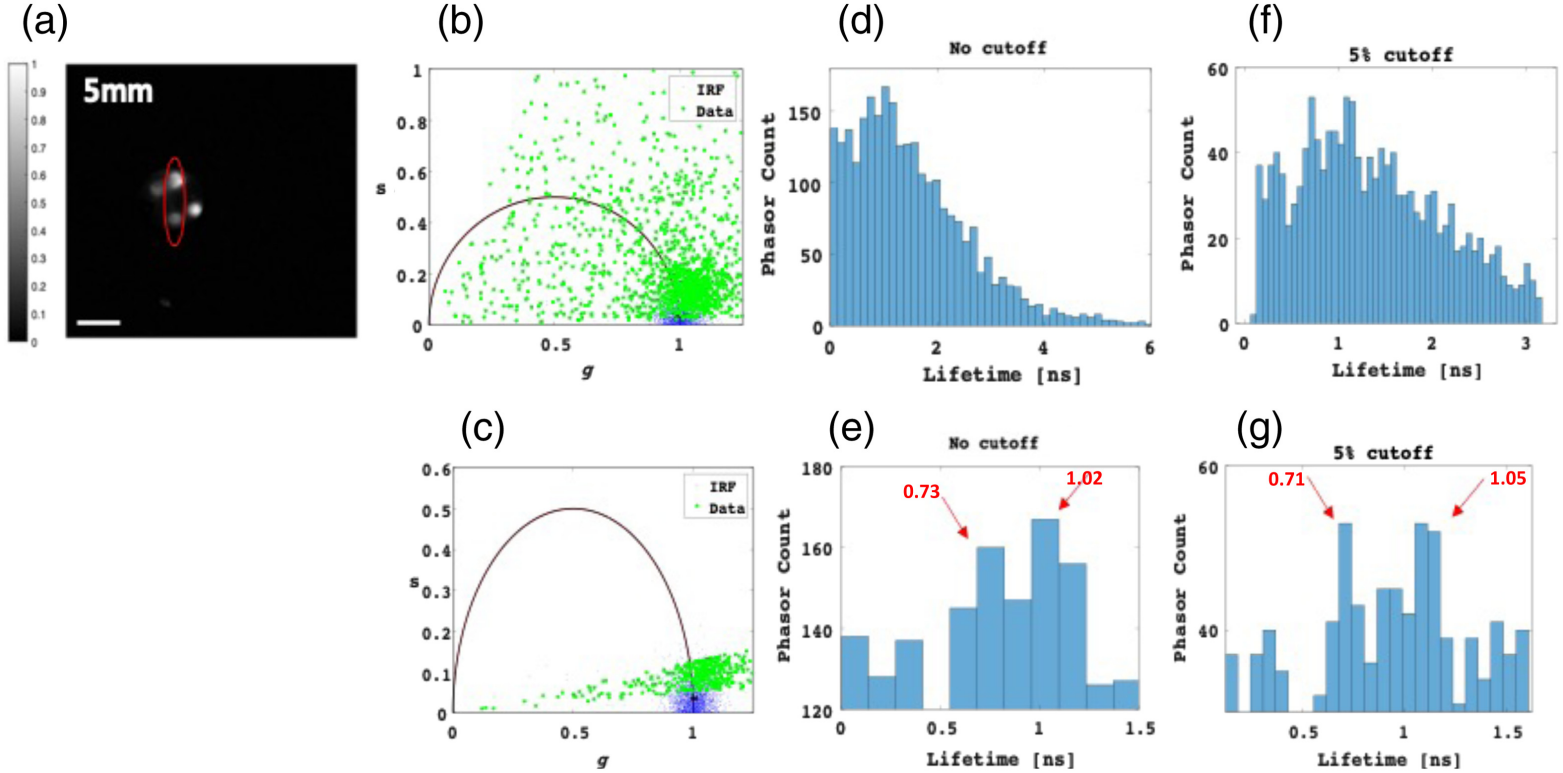
Multiplexing of ICG and IRDye800 behind a 0.5 cm IL tissue-like phantom. (a) Highlighted in red (ROI) are the dyes ICG and IRDye800. The scale bar measures 15 mm. (b), (c) Depiction of the phasor scatter plot for the ROI, without cutoff (b) and with a 5% cutoff (c). In the absence of a cutoff, the phasor points from the data display extensive scattering attributed to background noise. Implementing a 5% cutoff effectively eliminates a significant portion of this background noise. (d)–(g) Histograms detailing the distribution of phasor counts in relation to calculated lifetimes, obtained from each individual phasor point, for varying applied cutoffs. Specifically, (d) and (f) encompass the entire phasor histogram, while (e) and (g) zoom in on the 0 to 1.5 ns ROI where the expected FLTs of the dyes are anticipated.

After establishing our method for single-dye FLI behind tissue-like phantoms, we extended its application to the multiplexing of two adjacent dyes. Figure 8 shows the multiplexing capability of our phasor-based approach for the two dyes excited behind a 0.5 cm thick phantom. We analyzed the phasor FLTs within the pixelated area of the two dyes, indicated by the red ROI in Fig. 8(a). In this context, the region also contains a background (the dark space between the two prominent spots), which result with a very dispersed phasor “cloud” originating from the background within the ROI, as shown in Fig. 8(b). Consequently, discerning the FLTs of the two dyes becomes challenging. However, by selecting a suitable time range where the expected FLTs lie [e.g., 0 to 1.5 ns, as depicted in Fig. 8(e)], we successfully extracted the FLTs using a cutoff of 5% only (using a 1% cutoff did not yield significantly improved results; the resulting histogram showed no noticeable enhancement, as the background and dark noise is higher), resulting in a notably less scattered phasor cloud, exhibiting a marked reduction in background noise. The phasor histogram now exhibits two distinct Gaussian distributions, each aligning with the individual FLT of the respective dye [Fig. 8(g)]. These results underscore the fact that, whether utilizing the cutoff approach or not, by adeptly selecting the appropriate time range within the phasor histogram, we can effectively achieve multiplexing of FLTs for the two dyes present in the intensity images.

**Fig. 9:**
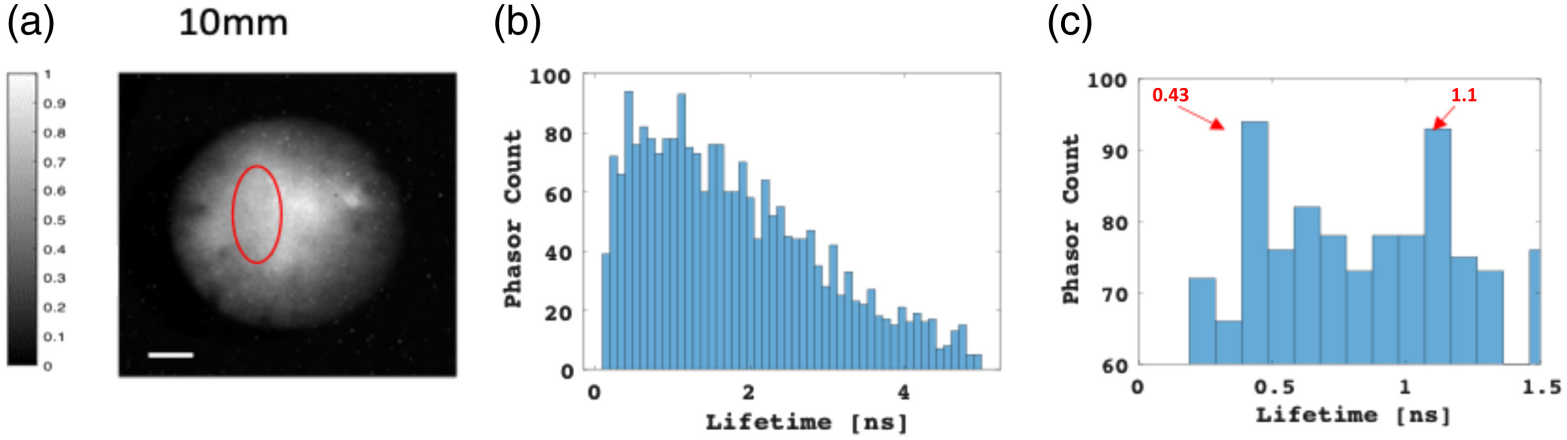
Multiplexing of ICG and IRDye800 behind a 1 cm thick IL tissue-like phantom. (a) Highlighted in red (ROI) are the fluorescence emissions of ICG and IRDye800. The scale measures 15 mm. (b) Phasor histograms of the circled ROI after applying cutoffs of 5%. (c) Histograms illustrating the phasor counts juxtaposed with the calculated lifetimes from each individual phasor point. This depiction focuses on the 0 to 1.5 ns time range of interest, where the anticipated FLTs of the dyes are expected.

Similar analyses were conducted on data acquired while imaging through a phantom with a thickness of 1 cm. Figure 9 shows phase analyses performed on the region delineated in red, corresponding to 5% cutoff in percentages. The outcomes highlight that when employing a 1 cm thick phantom, the tallest columns correspond to FLTs of 0.6 and 1.05, aligning with the FLTs of ICG and IRDye800, respectively.

The combination of the SPAD512S’s single photon detection capabilities alongside phasor-based analyses points toward the straightforward feasibility of multiplexing two adjacent dyes, following a single wavelength excitation. These time-gated, wide-field FLI findings underscore that in scenarios where the fluorescence signal significantly surpasses the autofluorescence from the phantom, the sample’s fluorescence lifetime can be accurately determined.

Important to note, the variations in the FLT of ICG (while the IRdye exhibited a relatively stable FLT in both, 5 and 10 mm phantoms) may stem from the higher impact of the phantom layer on the background noise, when ICG was imaged behind a 1 cm in thickness phantom. Although we subtract the phantom layer as a background, there is some inhomogeneity in the phantom layer which leave some influence of the phantom layer on the resulted FLT image. Despite our efforts to consistently stir the phantom solution during preparation, the potential for inhomogeneity arises when the phantom solidifies. The phantom itself possesses a very low FLT (see Ref. 7), and consequently, when its impact on the sample is significant, the FLT of the sample might decrease, as exemplified in ICG behind a 1 cm thickness phantom.

## 4 Discussion and Conclusions

Investigating the capabilities of FLI for multiplexing different dyes holds significant importance in the realm of biomedical imaging and diagnostics. Fluorescence lifetime, being an intrinsic property of fluorophores, offers a unique and quantitative dimension to fluorescence-based techniques. By effectively distinguishing dyes with similar emission spectra through their distinct fluorescence lifetimes, the multiplexing approach enhances the information content that can be extracted from a single image. This has profound implications across various fields, from cancer research to drug delivery and molecular imaging. Understanding the intricate interplay between fluorescence lifetimes, QYs, and tissue properties empowers researchers to design more accurate and informative imaging strategies, enabling the simultaneous detection and characterization of multiple targets within complex biological and clinical samples.

The NIR range of the electromagnetic spectrum is particularly appealing for clinical fluorescence-based imaging due to the diminished autofluorescence and scattering of tissue, coupled with the emergence of highly sensitive, noninvasive, and quantifiable FI techniques. Consequently, instruments tailored for wide-field FLI, imperative for *in vivo* measurements, have been developed over the last few decades.^29^^–^^33^ Among the most notable and extensively employed tools for *in vivo* FLI are the time-gated SPAD cameras,^34^^,^^35^ which have found widespread application in wide-field FLI.^30^^,^^36^ For instance, Ulka et al. introduced the SwissSPAD2, a substantial time-gated SPAD camera sporting 512×512  pixels. Demonstrating its capabilities in the visible range, they extracted FLTs of organic dyes using phasor analysis. Their subsequent study revealed SwissSPAD2’s consistent and high-quality imaging prowess for biologically relevant fluorescent samples stained with multiple dyes across varying conditions.^37^ They delved into factors, such as gate length, the number of gates, and signal intensity’s influence on the precision and accuracy of measured lifetimes. The integration of distinct species into a single image is facilitated by the expansive capabilities of large-format SPAD cameras. Multiplexing FLI of diverse targets within a single image represents a compelling research domain brimming with numerous potential applications.^38^^,^^39^ For instance, Chen et al. embarked on a study of FRET kinetics, both *in vitro* and *in vivo*, utilizing a time-gated camera with the phasor analysis software tool, AlliGator.^40^ Their findings on IgG binding kinetics underscored that both conventional matching and phase analysis yielded equivalent qualitative trends. Furthermore, they validated the phasor technique as an alternative for lifetime matching, showcasing *in vivo* FRET kinetics in bladder and liver tissues. In another notable research, phasor analysis demonstrated its utility in extracting fluorescence lifetime times (FLTs) from mixtures of NIR dyes in both *in vitro* and *in vivo* FRET contexts.^41^ Smith et al. took this exploration a step further, presenting a macroscopic NIR *in vivo* measurement termed FLI-FRET (MFLI-FRET) utilizing SwissSPAD2.^8^ Their investigations spanned from *in vitro* assessments focused on monitoring fluorescence lifetime variations due to environmental factors, to the quantification of FRET interactions between dyes. Notably, they successfully expanded these explorations to *in vivo* studies centered around FRET.

Despite the extensive work centered on time-dependent phasor-based FLI encompassing *in vitro* and *in vivo* scenarios, none of these studies delved into a comprehensive exploration of the method’s multiplexing capabilities as influenced by dye QY and tissue thickness, elements we comprehensively investigate in this study. We initiate by introducing a novel FLI simulation model, grounded in simplified assumptions implemented via rapid MC simulations targeting clinically dyes characterized by diverse lifetimes and QYs. This model systematically outlines the conduct of fluorophores at varying depths within a scattering medium, followed by the extraction of simulated dye FLT using the phasor technique. Our endeavor proceeds to showcase FLI of two neighboring dyes situated at distinct spatial separations and depths. This approach establishes a framework for effortlessly multiplexing two targets within a single image. Further exploration warrants an in-depth examination of the relationship between quantum efficiency and the count distribution within the lifetimes histogram for adjacent dyes. Across the two evaluated parameters, tissue depth and vertical distance between dyes, it was consistently observed that the greatest count frequency within the lifetime histogram corresponded to the shorter lifetime. This persisted even when the FLT aligned with the dye possessing the lowest quantum efficiency. This outcome defies initial expectations, as it was anticipated that the dye with the inferior quantum efficiency would emit fewer photons. This intriguing observation, though not influencing the efficacy of our method for FLT extraction, remains an area for potential further investigation in our future undertakings.

It is important to mention nonfitting methods for lifetime extraction, which were well demonstrated in recent published paper.^42^^,^^43^ Thus, e.g., Lukina et al.^43^ developed a tool to identify optical markers distinguishing between tumors and healthy tissues, utilizing a macroscopic FLIM. Employing FLIM in conjunction with novel non-fitting algorithms, they identified imaging markers for tumors. This method holds promise for glioma diagnostics and the assessment of surgical margins in gliomas. One key advantage of non-fitting FLI is their capacity to provide rapid and real-time measurements, eliminating the time-consuming computational processes associated with fitting models to experimental data. This not only accelerates data acquisition but also facilitates dynamic studies and enables the monitoring of fast biological processes. In addition, non-fitting methods are often less sensitive to experimental noise and are more robust in the presence of complex sample matrices, making them particularly suitable for *in vivo* or heterogeneous samples. However, one significant drawback of the non-fitting FLI methods is the lack of spatial resolution in delivering targeting to specific areas within cells. Fitting-based approaches, with their ability to discern distinct fluorescence lifetimes associated with different cellular components, allow for precise localization and analysis at the subcellular level. Non-fitting methods often struggle to provide detailed information on specific regions within a cell, limiting their utility in applications where spatial resolution is crucial, such as in targeted drug delivery or studying localized cellular processes. The absence of precise spatial information can hinder the accuracy and specificity required for understanding complex cellular dynamics, thereby restricting the scope of non-fitting FLI in certain research and clinical contexts.

Subsequently, we subjected our FLT extraction methodology to experimentation, employing the exquisitely sensitive time-triggered camera, SPAD512S. Our analyses effectively demonstrated the potential for multiplexing FLI, even when faced with a 1 cm thick scattering phantom layer. Our experimental outcomes underscore the feasibility of extracting the FLT of a solitary dye from the FLT histogram by leveraging the histogram’s mean value, enabling the differentiation of two dyes within a single image. Opportunities for refining image composition through intensified focal plane illumination and the adoption of 3D imaging techniques, such as optical tomography, hold promise for enhancing our experimental outcomes. This augmentation should contribute to bolstered resolution and a greater extent of tissue penetration.

In summation, the findings from our MC FLI phasor-based model and the subsequent validation through our expansive experimental wide-field FLI investigations collectively furnish an in-depth exploration of phasor-based FLI geared toward multiplexing objectives. The outcome is an innovative and straightforward avenue for the FLI of diverse targets seamlessly embedded within a singular frame.

## Supplementary Material



## Data Availability

Data underlying the results presented in this paper are not publicly available at this time but may be obtained from the authors upon reasonable request.
