# Supplementary material for: Multiplexed near infrared fluorescence lifetime imaging in turbid media
Source: J Biomed Opt. 2024 Feb 29;29(2):026004. doi: 10.1117/1.JBO.29.2.026004 (PMC10902792; doi:10.1117/1.JBO.29.2.026004)
Supplement: Supplementary file 1 [file JBO_029_026004_SD001.pdf]

Supplemental Material - *Multiplexed Near Infrared Fluorescence Lifetime Imaging in Turbid Media*

Meital Harel, Uri Arbiv and Rinat Ankri

Ariel University, Faculty of Natural Science, Department of Physics, Ariel, Israel

Content:

Figure S1. Emitted photons vs. time, for various fluorophores' photophysical characterization.

Figure S2. The sliced tissue-like phantoms were used for the FLI experiments

Figure S3. Average intensity <I> (measured in counts) for different depths z

Figure S4. Mean lifetime values for different fluorophores corresponding to diverse depths

Figure S5. intensity pictures of a single fluorophore located at a representative depth of z=0.3 cm

Figure S6. Representative lifetime results for intensity pictures presented in Fig. 2.

Figure S7. Simulated fluorescence intensity and phasor analysis results, with higher scattering coefficient

Figure S8. Simulated fluorescence intensities and lifetime histograms for two adjacent fluorophores

Figures S9 and s10. Simulated fluorescence intensity and lifetime histogram with 1% cutoff for two adjacent fluorophores

Figure S11. Extracted lifetimes versus depth:

Figure S12. The decay profiles of ICG fluorescence emission vary across different phantom slices.

Figure S13: Extracting FLTs of IRDye800 imaged through intralipid tissue-like phantoms with thicknesses varying from 0 to 1cm.

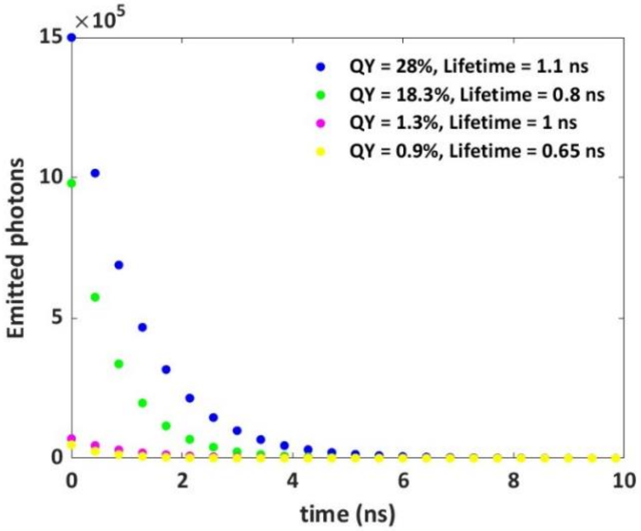

Figure S1. Emitted photons vs. time, for various fluorophores' photophysical characterization: (i) Quantum Yield (QY) = 28% Lifetime = 1.1 ns (blue). (ii) QY = 18.3%, Lifetime = 0.8 ns (green). (iii) QY = 1.3% Lifetime = 1 ns (magenta). (iv) QY = 0.9% Lifetime = 0.65 ns (yellow). All exponential decays started with the same initial photons number: 5*106. Time step between two dots is $g_s = 428\ ps$, while there are 117 dots (only the first 24 are presented in Figure S1).

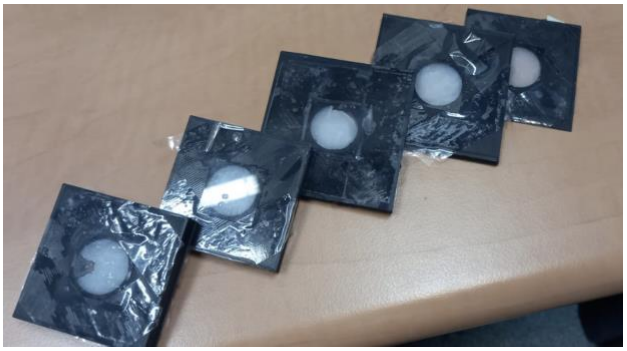

Figure S2. The sliced tissue-like phantoms were used for the FLI experiments. For each experiment, a slightly oversized slice of phantom was cut and placed on a phantom holder comprised of a glass coverslip bottom taped to the 3D printed spacer of appropriate thickness (0.1, 0.3, 0.5, 0.7 and 1 cm), then cut with a knife to achieve the desired thickness, and covered with another coverslip.

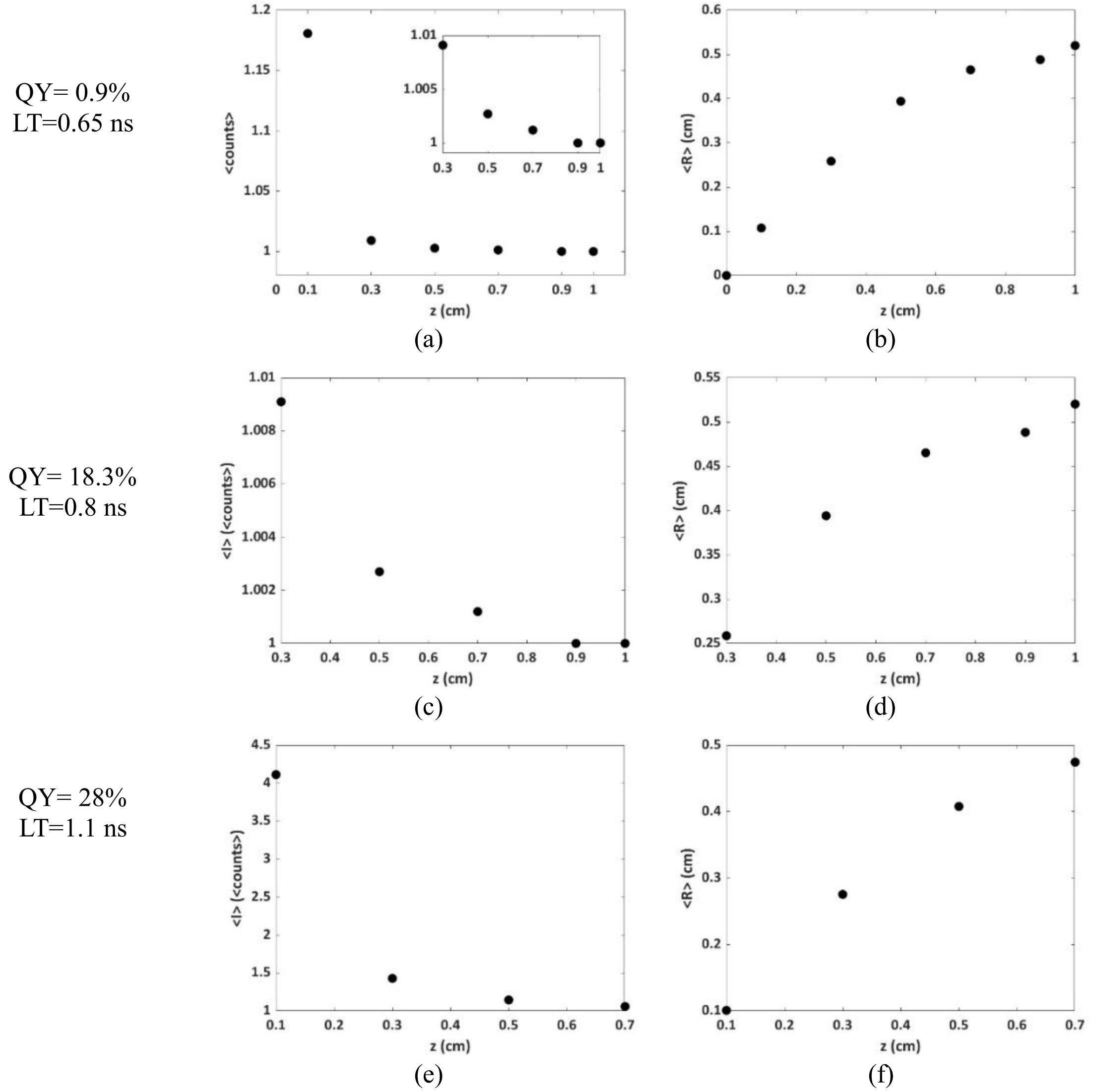

Figure S3. Average intensity <I> (measured in counts) for different depths z (panels a, c, e), as well as the average radius <R> as a function of z (panels b, d, f) for three fluorophores with optical properties presented in the left column.

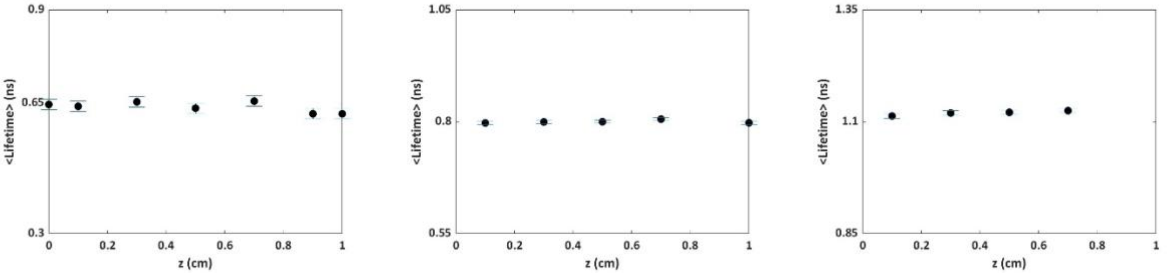

(a) (b) (c)

Figure S4. Mean lifetime values for different fluorophores corresponding to diverse depths while the ground true values are (a) 0.65 ns, (b) 0.8ns and (c) 1.1 ns.

Figure S4 exhibits mean lifetime values for the fluorophore with different lifetimes corresponding to diverse depths (IRDye800 is presented in Fig. 3f in the paper). The computed mean lifetime versus depth yields a value of 0.65 ± 0.01 ns, 0.79 ± 0.00 ns, and 0.1.11 ± 0.00 ns across all tissue depths, while the ground true values are 0.65 ns, 0.8ns and 1.1 ns, respectively.

(a) QY = 0.9% lifetime = 0.65 ns

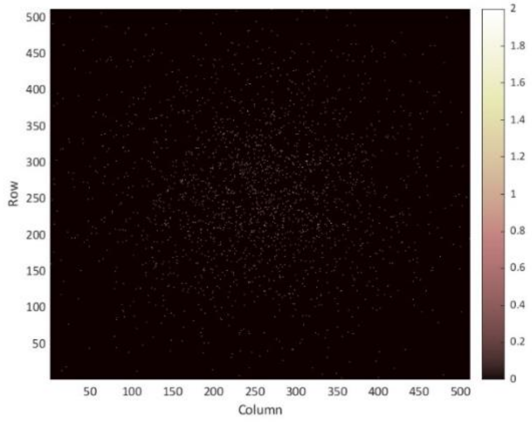

(b) QY = 1.3% lifetime = 1

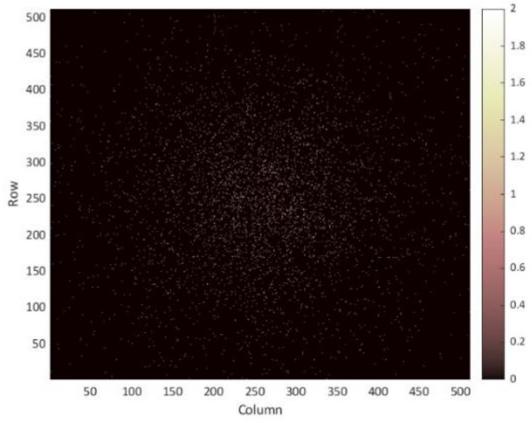

(c) QY = 18% lifetime = 0.8 ns

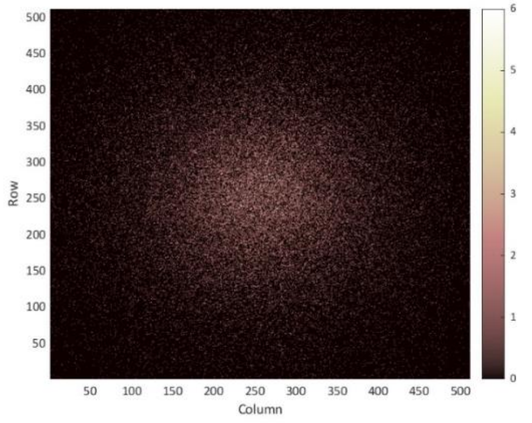

(d) QY = 28% lifetime = 1.1 ns

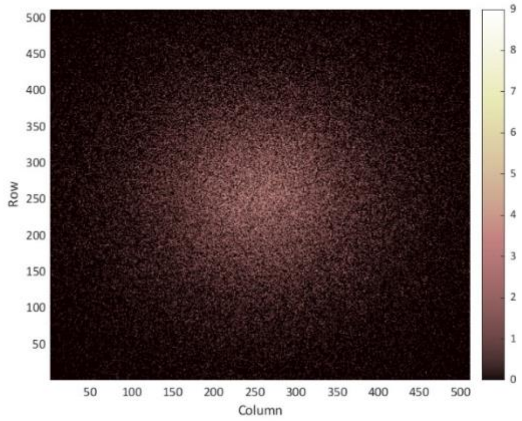

Figure S5. intensity pictures of a single fluorophore located at a representative depth of z=0.3 cm within the simulated tissue. (a) QY = 0.9% lifetime = 0.65 ns. (b) QY = 1.3% lifetime = 1. (c) QY = 18% lifetime = 0.8 ns. (d) QY = 28% lifetime = 1.1 ns.

Figure S6 shows two representative lifetime results for the intensity pictures presented in Fig. 2. These lifetime results are the foundation for the graph presented in Fig. 3f.

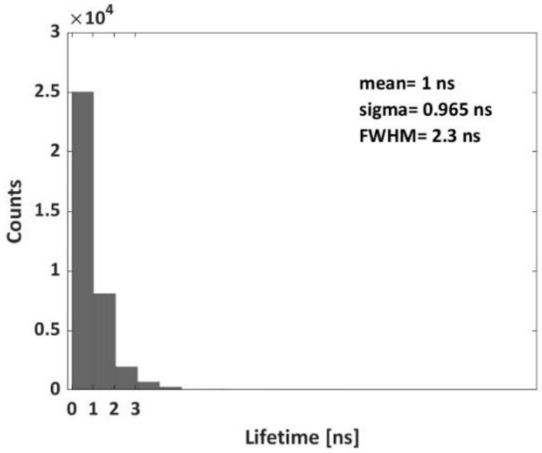

(a) Z=0.1 cm

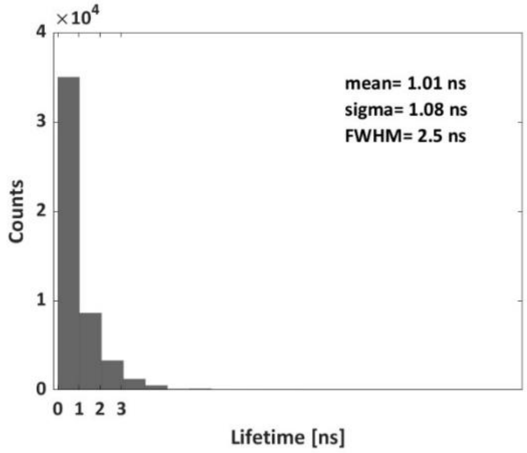

(b) Z=0.3 cm

Figure S6. Representative lifetime results for intensity pictures presented in Fig. 2. These lifetime results are the foundation for the graph presented in Fig. 3f.

We conducted simulations involving various optical properties, including a scattering coefficient that is an order of magnitude higher than the one presented in the paper (μs=503 cm-1 instead of μs=403 cm-1). The multiplexing results for the fluorescence lifetimes of the simulated samples at a depth of z=0.1 cm are shown in Figure S6. The simulations utilized phasor-based analyses for extracting lifetimes of two fluorophores: QY1 = 0.9%, τ_1 = 0.61 ns (left spot) and QY2 = 1.3%, τ_2 = 1.01 ns (right spot) with a fixed vertical distance Δx=2cm (Figure S6(a)). The region of interest (ROI) is indicated by the white rectangle. Figure S6(b) displays the lifetime histograms and phasor analyses for the entire image frame presented in Figure S6(a), while Figure S6(c) depicts the lifetime histograms and phasor analyses specifically for the selected ROI.

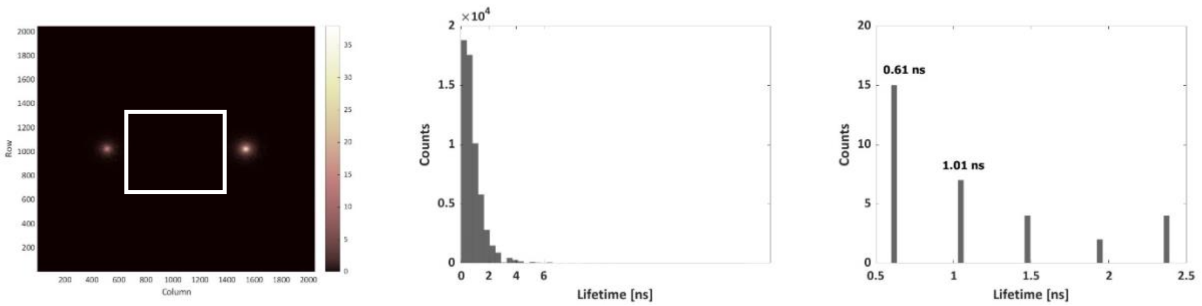

(a) (b) (c)

Figure S7. Simulated fluorescence intensity and phasor analysis results, with higher scattering coefficient μs=503 cm-1, pertaining to two adjacent fluorophores: Two adjacent fluorophores (left spot – QY=0.9%, lifetime = 0.65 ns, right spot - QY=1.3%, lifetime = 1 ns) from a top-down view. The vertical separation between the centers of these fluorophores was Δx = 2 cm, with an initial photons number a of $\sim 7.6 \cdot 10^7$. (a) The simulated intensity distribution, for a fluorophore's depth of 0.1 cm within the tissue. Dimensions are 2048*2048 pixels. The white square designates the ROI where phasor analyses were conducted (b) The histogram of lifetimes, for the large frame illustrated in Figure 1(a). (c) The histogram of lifetimes for the white frame of Figure 1(a), featuring a cutoff at 1%.

	Intensity pictures	LT histogramWith 1% cutoff

$\Delta x = 1\ cm$

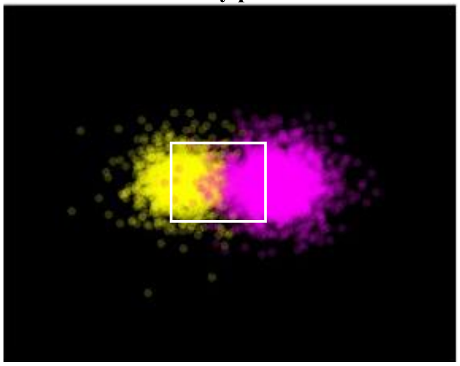

(a)

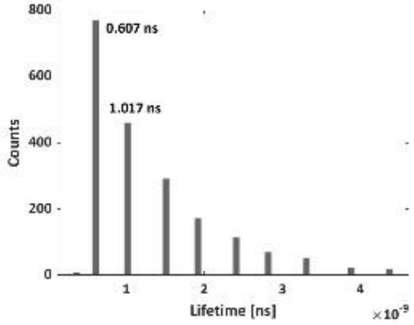

(c)

$\Delta x = 1.25\ cm$

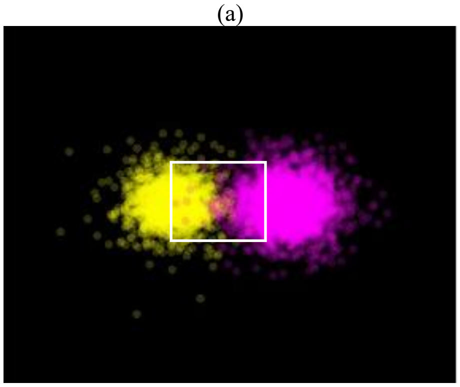

(f)

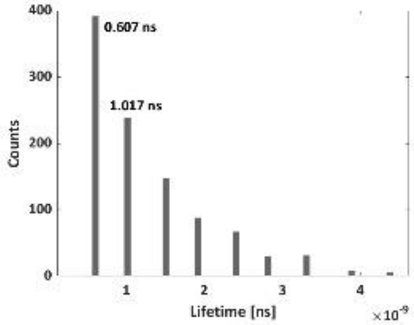

(h)

$\Delta x = 1.5\ cm$

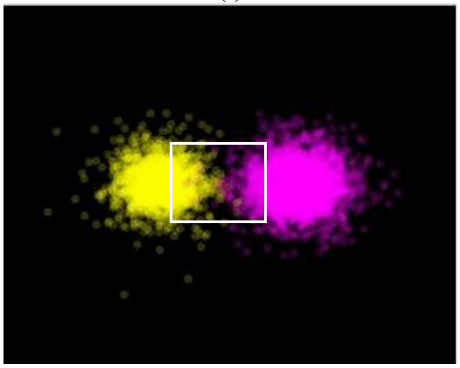

(k)

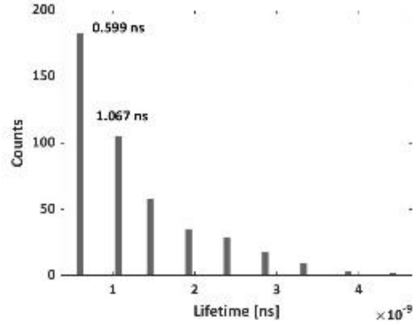

(m)

$\Delta x = 1.75\ cm$

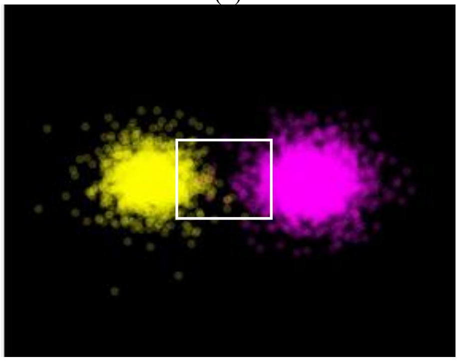

(p)

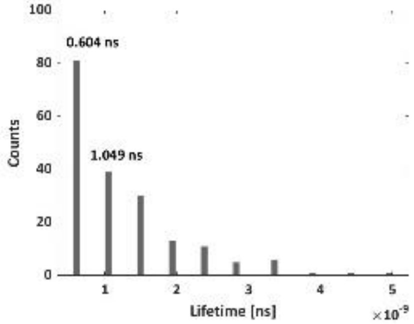

(r)

Figure S8. Simulated fluorescence intensities and lifetime histograms for two adjacent fluorophores with a fixed depth inside the tissue z = 0.3 cm that were placed in various vertical distance $\Delta x = 1, 1.25, 1.5, 1.75\ cm$**.** Lifetime histograms with 1% cutoff are presented as well.

z = 0

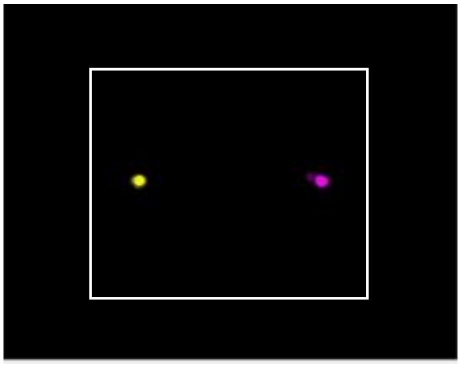

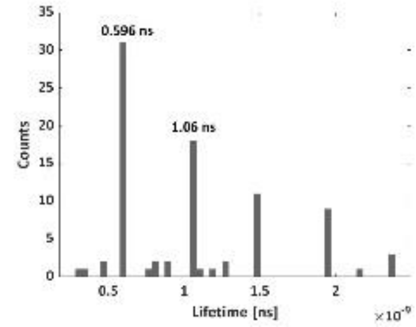

(a) (b)

z = 0.1 cm

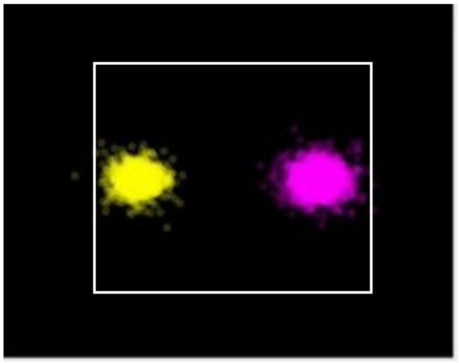

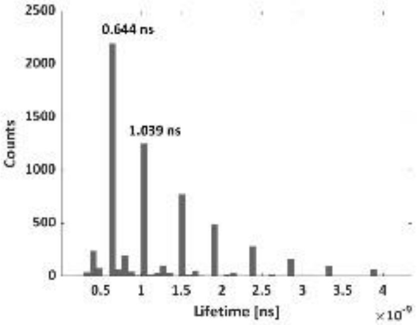

(c) (d)

z = 0.5 cm

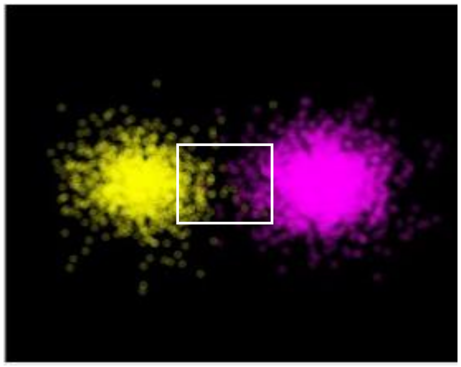

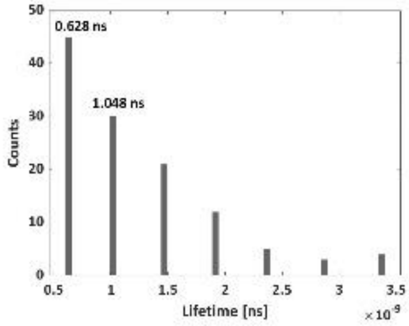

(e) (f)

z = 0.7 cm

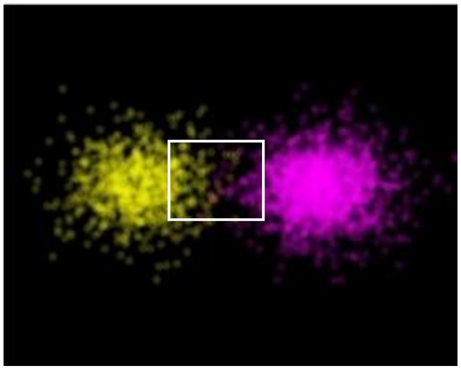

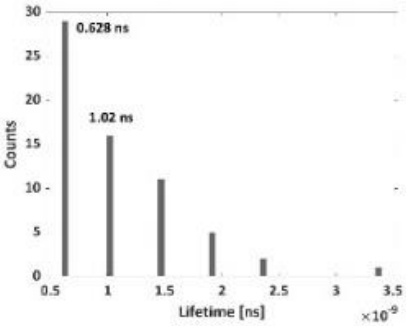

(g) (h)

z = 0.9 cm

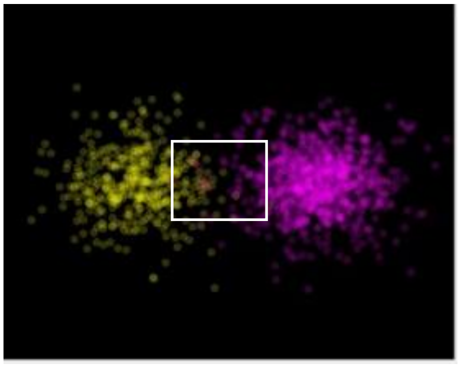

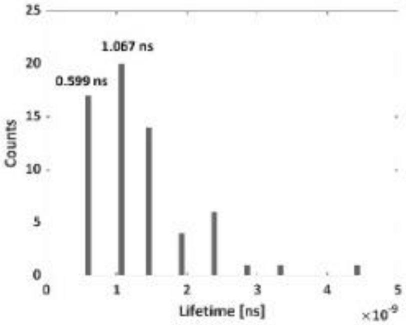

(i) (j)

z = 1 cm

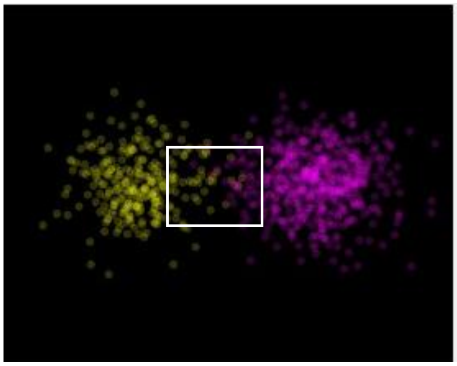

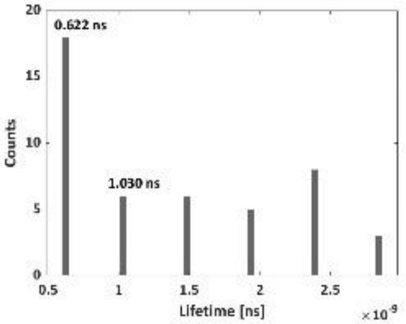

(k) (l)

Figure S9. Simulated fluorescence intensity and lifetime histogram with 1% cutoff for two adjacent fluorophores with a fixed vertical distance $\Delta x = 2\ cm$ that were placed various depths inside the tissue: (i) z = 0 – (vi) z = 1 cm**.** The two fluorophores presented the following optical properties: QY_1 = 0.9%, τ_1 = 0.62 ns (yellow spot) and QY_2 = 1.3%, τ_2 = 1 ns (magenta spot), with fixed separation distances, $\Delta x = 2\ cm$, as function of the tissues' thickness,

$z = 0{:}\,1cm$ are presented in Figure S6. (Results for z = 0.1,0.3 cm are presented in Figure 4 in the paper). The ROI (white frame) for z $= 0.5 - 1$ cm is 1 $cm \cdot 1$ cm , whereas the ROI for z $= 0 - 0.1$ cm is 3 $cm \cdot 3$ cm.

With 1% cutoff

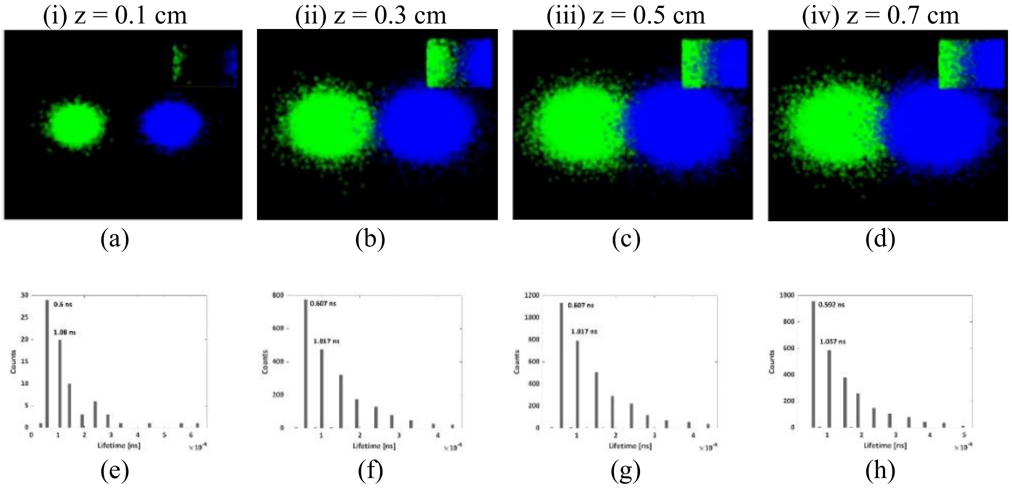

Figure S10. Simulated fluorescence intensities and lifetime histograms for two adjacent fluorophores with a fixed vertical distance $\Delta x = 2\ cm$, that were placed various depths inside the tissue: (i) z = 0.1 – (iv) z = 0.7 cm**.** Lifetime histograms with 1% cutoff are presented as well. The optical properties of the two fluorophores were : QY_3 = 18.3%, τ_3 = 0.8 ns (green spot) and QY_4 = 28%, τ_4 = 1.1 ns (blue spot), The ROI (inset) is $1\ cm \cdot 1\ cm$.

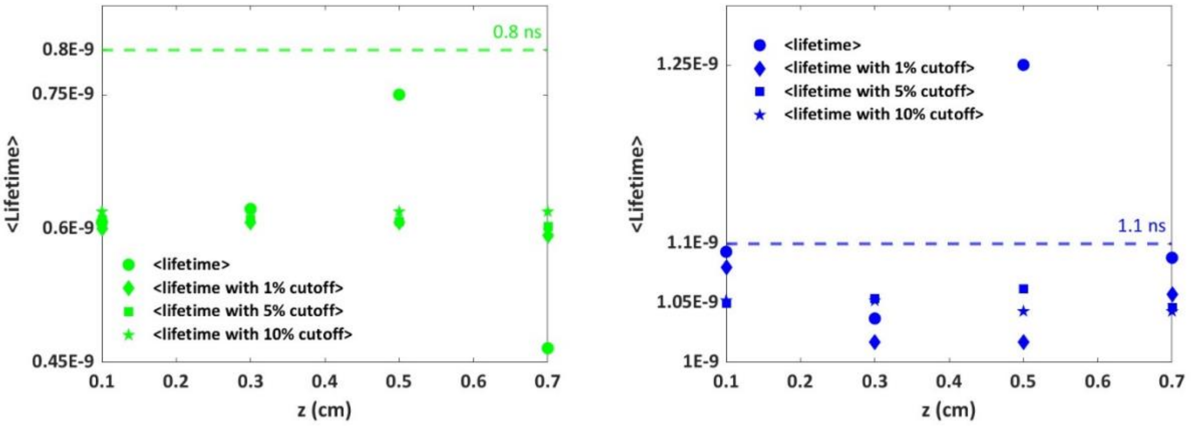

(a) (b)

Figure S11. Extracted lifetimes versus depth: without cutoff and with 1%, 5%, and 10% cutoff for two adjacent fluorophores with a fixed vertical distance $\Delta x = 2cm$. **(a)** The fluorophore F_3 **(b)** The fluorophore F_4. This figure summarizes the multiplexing results for the FLTs of the simulated samples (at the different depths $z = 0.1, 0.3, 0.5, 0.7\ cm$), without and with 1%, 5%, and 10% cutoff. The two fluorophores: F_3 – green (Figure S8(a)) and F_4 -blue (Figure S8(b))) had a fixed vertical distance $\Delta x = 2cm$.

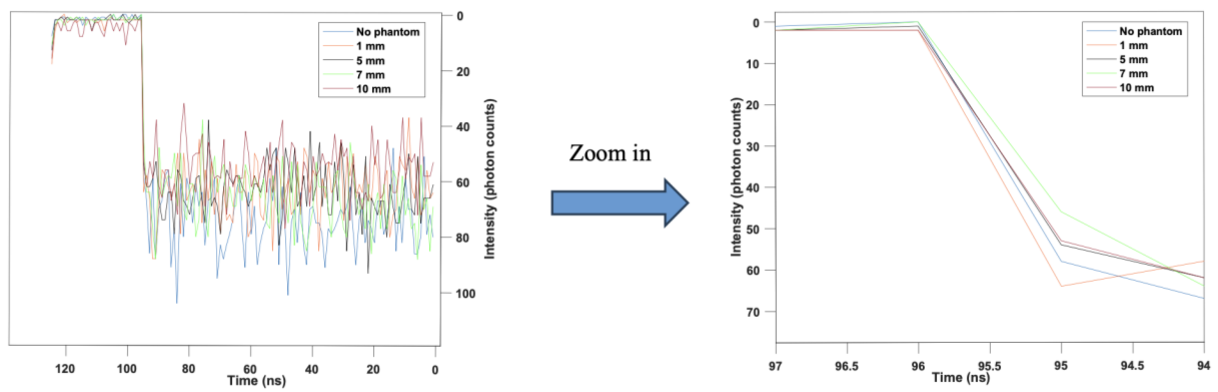

Figure S12. The decay profiles of ICG fluorescence emission vary across different phantom slices.

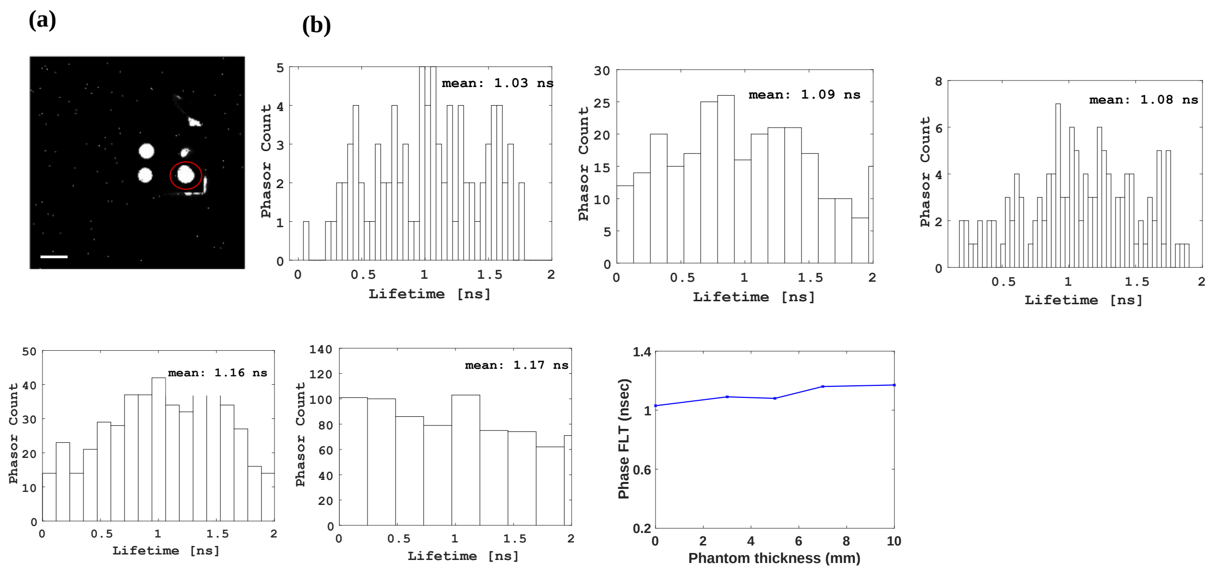

Figure S13: Extracting FLTs of IRDye800 imaged through intralipid tissue-like phantoms with thicknesses varying from 0 to 1cm. (a) The red circled dye is the IRDye800. Scale bar is 11mm. (b) Histograms for the phasor counts versus the lifetimes calculated from each phasor point. (d) The mean FLT for each phantom's thickness. (c) The mean FLT for each phantom's thickness. With the power of the microlensed SPAD512S, pixel-by-pixel background correction and phasor-based analyses, the FLT of the dye was extracted even behind a 1 cm phantom through the calculation of the mean of the phasor cloud.
